# Gut Microbiota‐Isoallolithocholic Acid Crosstalk Promotes Calcium Oxalate Kidney Stone Formation via PARP1‐Mediated Parthanatos

**DOI:** 10.1002/advs.77289

**Published:** 2026-08-28

**Authors:** Zijian Zhou, Ruixi Yu, Xuan Zhou, Feng Shi, Zeyu Lin, Peng Gao, Lujia Wang, Ninghan Feng, Zhong Wu, Dawei Wang

**Affiliations:** ^1^ Department of Urology Huashan Hospital Fudan University Shanghai P. R. China; ^2^ Clinical Research Center of Urolithiasis Shanghai Medical College Fudan University Shanghai P. R. China; ^3^ Department of Urology Jiangnan University Medical Center Wuxi P. R. China; ^4^ Precision Research Center for Refractory Diseases Department of Urology Shanghai General Hospital Jiaotong University School of Medicine Shanghai P. R. China; ^5^ Civil Aviation Shanghai Hospital Gubei Branch of Ruijin Hospital Shanghai P. R. China; ^6^ Department of Critical Care Medicine Nanjing First Hospital, Nanjing University of Chinese Medicine Nanjing P. R. China; ^7^ Wuxi School of Medicine Jiangnan University Wuxi P. R. China; ^8^ Department of Urology Ruijin Hospital School of Medicine Shanghai Jiao Tong University Shanghai P. R. China

**Keywords:** bile acid, calcium oxalate nephrolithiasis, gut microbiota, PARP1, parthanatos

## Abstract

Bile acids have been implicated in calcium oxalate (CaOx) nephrolithiasis. Here, we employ multi‐omics approaches to identify isoallolithocholic acid (isoalloLCA) as the key bile acid elevated in the feces, serum, kidney, and urine of CaOx rats, confirmed by spatial metabolomics. 16S sequencing and metagenomic analyses indicate that elevated isoalloLCA levels in CaOx rats or individuals are likely of microbial origin. Mechanistically, isoalloLCA binds to PARP1 via specific molecular interactions validated by surface plasmon resonance and cellular thermal shift assays. Knockdown of PARP1 by adeno‐associated virus microinjection or pharmacological inhibition with PARP1 inhibitor AZD5305 significantly attenuates isoalloLCA‐mediated crystal deposition, renal tubular injury, and mitochondrial functional impairment in both the CaOx rat and mouse models. Furthermore, the antibiotic‐mediated depletion of the gut microbiota in mice markedly reduced isoalloLCA levels, whereas fecal microbiota transplantationut contributes to isoalloLCA‐mediated CaOx stone formation, demonstrating a causal link between gut microbiota and isoalloLCA. In vitro, isoalloLCA promoted oxalate‐induced renal tubular epithelial cell injury and parthanatos via targeting PARP1, including DNA damage, excessive PARP1 activation, PAR accumulation, mitochondrial damage, nuclear translocation of AIF and MIF. These findings establish the microbiota‐isoalloLCA‐PARP1‐parthanatos axis in CaOx nephrolithiasis and identify PARP1 as a promising therapeutic target for kidney stone management.

## 1 Introduction

Kidney stones are a common urological disorder, with an increasing prevalence observed over the past few decades [1]. The presence of kidney stones can lead to renal colic, urinary tract infections, kidney damage, and even end‐stage renal disease [2]. Additionally, the high recurrence rate of kidney stones contributes significantly to the global public health burden [3, 4]. Approximately 80% of kidney stones are predominantly composed of calcium oxalate (CaOx) crystals [5]. While the etiology and pathogenesis of renal CaOx stones are complex and not fully understood, common metabolic factors contributing to CaOx formation include high urinary oxalate (hyperoxaluria) and calcium (hypercalciuria) levels, which lead to urine supersaturation of minerals [6].

The gut‐kidney axis refers to the interactions between the gut microbiota and the kidneys, where gut microbiota can influence kidney function and potentially lead to CaOx formation through metabolites, inflammation, and immune mechanisms [7]. For instance, gut microbes capable of degrading oxalate, such as *Oxalobacter formigenes*, can limit oxalate absorption and reduce oxalate excretion, thus impacting CaOx formation [8, 9]. Metabolites from certain gut microbiota, such as short‐chain fatty acids (SCFAs), may influence the expression of intestinal oxalate transporters like SLC26A6, thereby inhibiting CaOx stone formation [10].

By analyzing urinary or blood metabolomic profiles, researchers have identified characteristic metabolites associated with kidney stone formation, such as succinate, β‐cryptoxanthin, and sphingomyelin, which may provide a basis for early diagnosis and personalized treatment [11, 12]. Bile acids are key substances in the digestion of fats in the intestine, where they are metabolized by the gut microbiota into secondary bile acids, thereby becoming important mediators of metabolic homeostasis [13]. Recent studies have demonstrated that bile acids not only play a critical role in lipid digestion and absorption, but also significantly impact metabolism and inflammatory responses in kidneys [14, 15, 16]. Alterations in bile acid composition and levels might induce renal cell death, inflammatory responses, and fibrosis through the IRF3‐ZBP1 axis [15]. There are also theories that suggest bile acids may increase the colonic oxalate permeability, facilitating passive absorption of oxalate [17]. Moreover, our previous work found significant changes in the bile acid metabolism using non‐targeted metabolomics analysis in CaOx kidney stone rats [18]. Therefore, employing metabolomics to deeply analyze bile acids would offer new insights into the mechanisms of CaOx kidney stone formation.

Recently, multi‐omics analysis, integrating microbiome, metabolomics, spatial omics, transcriptomics, proteomics, and network pharmacology approaches, has been widely applied to study the pathogenesis and biomarkers of kidney diseases [19, 20, 21]. This comprehensive analysis not only elucidates the interactions among metabolites, genes, and proteins, but also identifies key biological regulatory factors, advancing personalized medicine and targeted therapies [22, 23]. However, existing studies focusing on bile acids or CaOx are often confined to a single omics layer, leaving our understanding of the complex relationships between bile acids and CaOx at a preliminary stage [24]. Therefore, we continued to employ targeted bile acid metabolomics in CaOx rat stool, serum, kidneys, and urine based on the gut‐kidney axis, and identified the key differential metabolites, isoallolithocholic acid (isoalloLCA), with spatial metabolomics of kidneys confirming this result. Furthermore, through proteomics and network pharmacology, we have identified PARP1 as the key target of isoalloLCA with employing experimental and computational approaches to investigate the interactions and binding mechanisms between isoalloLCA and PARP1 protein, and have validated their function and mechanism in vivo and in vitro, hoping to provide new insights for the prevention and treatment of CaOx kidney stones.

## 2 Methods

### 2.1 Establishing a CaOx Kidney Stone Model and Histological Analysis

A CaOx kidney stone model was developed using male Sprague‐Dawley rats, aged 5–6 weeks, with 1% ethylene glycol (EG) and 0.5% ammonium chloride (AC) in drinking water, which was approved by the Animal Ethics Committee of Fudan University. These 12 rats were divided into two groups: a control group (named Con), which had unlimited access to sterile tap water, and a model group (named CaOx), which consumed water containing 1% EG and 0.5% AC by gavage administration. After four weeks, the stool, serum, urine, ileal and renal tissues were collected for further analysis.

Ileal and renal tissues from rats were preserved using 10% formalin, embedded in paraffin wax, and subsequently sectioned into slices of 5 micrometers. Staining techniques were employed for kidney sections, including hematoxylin and eosin (H&E), Von Kossa (VK), alizarin red (AR), dihydroethidium (DHE), Masson, periodic acid‐schiff (PAS), and Terminal deoxynucleotidyl transferase dUTP Nick‐End Labeling (TUNEL) staining, following standard protocols. Immunohistochemical (IHC) analysis was conducted to assess the expression levels of kidney stone formation markers (CD44, MCP‐1, and OPN) using specific antibodies in the kidney sections. For the ileal sections, H&E and dual immunofluorescence (ZO‐1 and OCLN) staining were employed. The detailed information on the antibodies is listed in Table S1.

### 2.2 Enzyme‐Linked Immunosorbent Assay (ELISA)

The concentrations of interleukin‐1 beta (IL‐1β) and interleukin‐6 (IL‐6) in rat and mouse kidneys were quantified using specific ELISA kits designed for these cytokines, in accordance with the manufacturer's instructions. Briefly, kidney tissues homogenates were prepared and loaded into 96‐well plates pre‐coated with antibodies specific to IL‐1β or IL‐6. After incubation, unbound substances were thoroughly washed away, and enzyme‐conjugated secondary antibodies were added to bind the captured cytokines. Following additional incubation and washing steps, a substrate solution was added to facilitate a colorimetric reaction. The reaction was terminated, and the absorbance of each well was measured at 450 nm.

### 2.3 Targeted Bile Acid Metabolomics

For the solid samples, the stool and kidneys were processed by homogenizing in a methanol solution with 0.1% formic acid, followed by ultrasonic disruption and freezing at −20°C for 1 h. The samples were then centrifuged at 14 000g for 20 min to collect the supernatant. For the liquid samples, the serum and urine samples were treated by adding four volumes of cold 70% methanol/water containing 0.1% formic acid, vortexed, and centrifuged to obtain the supernatant. The LC/MS analysis was performed on a Waters ACQUITY UPLC HSS T3 C18 column using a gradient elution of water and acetonitrile, both containing 0.05% formic acid, over a 12 min cycle at a flow rate of 0.35 mL/min. Mass spectrometry was conducted using a QTRAP 6500+ system with ESI+/‐ settings, employing both linear ion trap and triple quadrupole scans. Statistical analyses were conducted using R software, applying Principal Component Analysis (PCA) and Orthogonal Partial Least Squares Discriminant Analysis (OPLS‐DA) with 200 permutation tests to curb overfitting. Discriminative metabolites were identified using variable importance in projection (VIP) values from OPLS‐DA, fold change (FC) and Student's t‐test on normalized data. Metabolites with VIP>1.0, |log_2_FC|>1, and *p* <0.05 were considered significant.

### 2.4 16S RRNA Sequencing

Fecal samples were analyzed using 16S rRNA sequencing to characterize the gut microbiota of rats. Sequencing libraries were prepared with the Illumina TruSeq kit and sequenced on the NovaSeq platform. OTU clustering was performed using Uparse, with species annotation through the SILVA and Unite databases. Alpha diversity, which measures the diversity within a sample, was assessed using various indices, including observed OTUs, Chao1, Shannon, and Simpson indices. Beta diversity analysis, including Principal Component Analysis (PCA), Principal Coordinate Analysis (PCoA) and Nonmetric multidimensional scaling (NMDS), was performed using QIIME, and UPGMA clustering helped interpret the distance matrices. Linear discriminant analysis Effect Size (LEfSe) is used to identify differentially abundant taxa between groups by first performing non‐parametric statistical tests to detect features with significant differences, followed by linear discriminant analysis (LDA) to estimate the effect size of each differentially abundant feature.

### 2.5 Clinical Samples and Metagenomic Sequencing

In this study, 20 patients diagnosed with CaOx nephrolithiasis (named CaOx group) were recruited from the Department of Urology at Huashan Hospital, Fudan University, along with 20 healthy controls (HC group). All participants met strict eligibility criteria, including no gastrointestinal infections or antibiotic use within the three months prior to enrollment. CaOx stone patients were identified during routine clinical visits prior to undergoing ureteroscopy or percutaneous nephrolithotomy, with stone composition analysis confirmed postoperatively. Healthy controls were selected from the community and frequency‐matched for age, sex, and body mass index (BMI). Stool samples were collected from all participants for metagenomic sequencing and targeted bile acid metabolomics analysis. Metagenomic sequencing libraries were constructed from 1 µg of DNA per sample using the NEBNext Ultra DNA Library Prep Kit (Illumina). DNA was fragmented to ∼350 bp by sonication, followed by end repair, adapter ligation, and PCR amplification. Library size and concentration were assessed with an Agilent 2100 Bioanalyzer and qPCR. Sequencing was performed on an Illumina NovaSeq platform. For species annotation, unigene sequences were aligned against the NCBI NR database using DIAMOND.

### 2.6 Spatial Metabolomics

Frozen tissue sections (10 µm thick) were prepared using a Leica CM1950 cryostat at −20°C and mounted on indium tin oxide (ITO)‐coated slides, followed by vacuum desiccation for 30 min. Matrix application was performed using a sprayer with 15 mg/mL DHB (2,5‐dihydroxybenzoic acid) in 90% acetonitrile, 10% water, at 60°C, 0.1 mL/min flow rate, and 6 psi pressure, with 20 passes and 5 s drying intervals. MALDI‐timsTOF MSI (Bruker timsTOF flex) was conducted in positive ion mode over a mass range of m/z 50–1300 Da. The spatial resolution was set to 50 µm, which represents the pixel size of the imaging raster and enables visualization of metabolite distributions at near‐cellular resolution. A total of 400 laser shots were accumulated per pixel, and the laser repetition rate was 2000 Hz. Spectra were normalized by root mean square (RMS) intensity to correct for pixel‐to‐pixel variation in ionization efficiency. Metabolite identification was confirmed by MS/MS fragmentation with collision energy set to 30–45 eV, matching fragment spectra against public databases (HMDB, METLIN). Image reconstruction and data visualization were performed using SCiLS Lab software (Bruker Daltonics), where ion intensity heatmaps were overlaid with optical images for precise anatomical correlation.

### 2.7 Proteomics

Rat serum and kidney tissues were collected and lysed using radioimmunoprecipitation assay (RIPA) buffer on ice, followed by sonication for further disruption. The lysates were then centrifuged to isolate proteins from the supernatant. Proteins were resolved using SDS‐PAGE electrophoresis, and the distinct bands were excised for trypsin digestion. Protein identification and quantification were performed using LC‐MS/MS. The resulting mass spectrometry data were analyzed using MaxQuant software, which facilitated peptide matching, protein identification, and quantitative analysis. This approach enabled the comparison of protein expression levels across samples, highlighting proteins with significant expression differences. Proteins with significant differences in expression between the control group and the CaOx stone group were selected using *p* < 0.05 and |log_2_FC| > 1. Gene Ontology (GO) and Kyoto Encyclopedia of Genes and Genomes (KEGG) pathway analysis was performed using Fisher's exact test with FDR correction, setting the significance threshold at *p* <0.05.

### 2.8 Network Pharmacology to Predict the Target of isoalloLCA in CaOx Kidney Stones

The molecular structure of isoalloLCA is sourced from the PubChem database (https://pubchem.ncbi.nlm.nih.gov/). Then, the SDF file of the three‐dimensional structure of isoalloLCA is uploaded to the PharmMapper database (http://lilab.ecust.edu.cn/pharmmapper/) to identify predicted human protein targets associated with isoalloLCA. Finally, the predicted protein sequences are converted into corresponding gene symbols using the UniProt database (https://www.uniprot.org/). As a result, a total of 292 pharmacological targets of isoalloLCA were screened out. Subsequently, 106 genes related to CaOx kidney stones were obtained from the Comparative Toxicogenomics Database (CTD, https://ctdbase.org/). A Venn diagram was used to display the common genes shared among PharmMapper, DEPs in rats, and CaOx‐associcated genes in the CTD database, leading to the identification of the potential target (PARP1) for isoalloLCA in CaOx kidney stones.

### 2.9 Surface Plasmon Resonance (SPR)

The binding affinities between isoalloLCA (200 µm) and the PARP1 protein were determined using a biosensor‐based SPR assay performed on a Biacore T200 instrument (Cytiva, Sweden). Recombinant Human PARP1 Protein (cat#11040‐H08B, Sino Biological, China) was diluted to a final concentration of 0.3 mg/ml using the standard amine coupling method [25]. Immobilization levels of PARP1 protein on the flow cell at 12 000 RUs. Test samples of isoalloLCA were dissolved in DMSO to prepare solutions at 200 μΜ. The SPR assay used PBST buffer with 1% DMSO, and all isoalloLCA samples were adjusted to this concentration. Data were processed with automatic reference subtraction and double background subtraction to eliminate non‐specific binding and system noise. The samples were injected over the sensor surface at a flow rate of 30 µL/min for 90 s, followed by running buffer for an additional 180 s to allow dissociation of the test sample from the immobilized protein. BIACore simulation of the SPR raw spectra provided corresponding association dissociation constant (Kd), and affinity values (KD).

### 2.10 Microscale Thermophoresis (MST)

The interaction between PARP1 protein and isoalloLCA was evaluated using MST on a NanoTemper Monolith NT.115 instrument. PARP1 protein was used as the target and fluorescently labeled with the Monolith Protein Labeling Kit RED‐tris‐NTA (MO‐L018, NanoTemper Technologies). The MST assay used a final DMSO concentration of 5% across all samples and isoalloLCA served as the ligand. For each assay, the labeled PARP1 protein (200 nm) was incubated with varying concentrations of isoalloLCA (ranging from 1000 to 0.0305 µm) in MST buffer at room temperature for 15 min. MST measurements were performed at 25°C using 100% LED excitation power and 40% MST power. The Kd value was calculated using MO Affinity Analysis V2.3 software.

### 2.11 Cellular Thermal Shift Assays (CETSA)

HK‐2 and NRK‐52E cells were incubated with either DMSO or isoalloLCA (200 µm) for 24h. Subsequently, the samples were subjected to heat treatment at various temperatures (37°C–6°C) for 3 min, followed by centrifugation at 12 000 rpm for 10 min at 4°C. The supernatants were collected and analyzed via western blot.

### 2.12 Molecular Docking and Molecular Dynamics (MD) Simulation

For molecular docking, we used AlphaFold‐predicted structures for both human PARP1 (UniProt P09874) and rat PARP1 (UniProt P27008) obtained from the AlphaFold Protein Structure Database (https://alphafold.ebi.ac.uk/). The 3D structure of isoalloLCA (Compound CID: 94228) was obtained from PubChem (https://pubchem.ncbi.nlm.nih.gov/). Perform molecular docking using isoalloLCA as the ligand with AutoDock Vina, and visualize the structures using PyMOL. MD simulations were performed using the AMBER22 software. The protein‐ligand complex was solvated in a dodecahedral water box with a 1.2 nm radius, and counterions were added to neutralize the system. Energy minimization and equilibration were conducted under NPT conditions to stabilize the system at 300 K and 1 bar. A total of 100 ns of production MD simulations were carried out, saving snapshots every 10 ps, resulting in 10 000 frames. CPPTRAJ was used for trajectory analysis, while binding free energy calculations were performed using the MMPBSA.py tool.

### 2.13 Microinjection of Adeno‐Associated Virus Into Rat Kidneys

To knockdown PARP1 expression in vivo, an adeno‐associated virus (AAV) serotype 9 vector encoding short hairpin RNA targeting PARP1 (shPARP1) or a negative control (shNC) were used. The sequence used to target the PARP1 was as follows: 5‐GAGTACATTGTCTACGACATT‐3. Three week before the CaOx modeling process, AAV (1×10^12^ vector genomes/mL; 10 µL) was micromicroinjected into the rat kidney at five distributed sites under direct visualization. Briefly, rats were anesthetized with isoflurane and placed in the lateral position. After sterilization and a dorsal incision, the kidney was exposed and stabilized. The renal capsule was punctured using an insulin needle, and AAV was slowly injected into renal parenchyma at five distributed sites. The actual total volume delivered was 50 µL, corresponding to a total viral load of 5 × 10^10^ vector genomes per kidney. The kidney was repositioned, and the incision sutured. Then, these rats were given standard diet or a diet containing 0.1% isoalloLCA for 4 weeks during the CaOx modeling process. The specific groupings and experimental arrangements are as follows: 1. The control rats were fed with a standard chow diet and microinjected with AAV‐shNC (Con+shNC group, n = 6); 2. The CaOx rats were fed with a standard chow diet and microinjected with AAV‐shNC (CaOx+shNC group, *n* = 6); 3. The CaOx rats were fed with a standard chow diet and microinjected with AAV‐shPARP1 (CaOx+shPARP1 group, *n* = 6); 4. The CaOx rats were fed with a 0.1% isoalloLCA diet and microinjected with AAV‐shNC (CaOx+isoalloLCA+shNC group, *n* = 6); 5. The CaOx rats were fed with a 0.1% isoalloLCA diet and microinjected with AAV‐shPARP1 (CaOx+isoalloLCA+shPARP1 group, *n* = 6). At the end of this experiment, rats were euthanized, and blood and kidney tissue samples were collected for renal function and morphology analysis.

### 2.14 Induce Kidney Stone Models in Mice via Glyoxylic Acid Intraperitoneal Injection

Male C57BL/6 mice weighing 20–25 g, aged 6–8 weeks, were randomly divided into four groups with five mice in each group. The model group received intraperitoneal injections of glyoxylic acid (75 mg/kg/day, cat#HY‐79494, MedChemExpress, USA) for 14 consecutive days (named Gly group), while the control group was injected with an equal volume of normal saline (named Con group). Additionally, the intervention group (named isoalloLCA group) was designed to examine the effects of isoalloLCA (cat#HY‐B0172A, MedChemExpress, USA) and the effects of PARP1 with PARP1 selective inhibitor AZD5305 (1 mg/kg/d, cat#HY‐132167, MedChemExpress, USA) on kidney stones (named AZD5305 group) [26, 27]. For the isoalloLCA group, the basic mice feed was an AIN‐93G standard rodent diet, whereas the diet of the isoalloLCA group was based on the AIN‐93G formula supplemented with an addition of 0.1% isoalloLCA. The specific groupings and experimental arrangements are as follows: 1. The mice of control group were fed with a standard chow diet, with the same volume of normal saline by intraperitoneal injection and gavage (Con group, *n* = 5). 2. The mice of the CaOx group were fed with a standard chow diet with glyoxylic acid intraperitoneal injection, with the same volume of normal saline gavage (CaOx group, *n* = 5). 3. The mice of isoalloLCA were fed a 0.1% isoalloLCA diet with glyoxylic acid intraperitoneal injection, with the same volume of normal saline gavage (isoalloLCA group, n = 5). 4. The mice of the AZD5305 group were fed a 0.1% isoalloLCA diet with glyoxylic acid intraperitoneal injection, with AZD5305 gavage (1 mg/kg/d). The injection volume did not exceed 0.2 mL per mouse, and injections were administered once daily for 14 consecutive days. After the completion of the injections, the mice were euthanized. The kidneys were rinsed with normal saline, fixed in 10% neutral formalin. Micro‐CT imaging was conducted using the SkyScan 1176 system (Bruker, Belgium) with a resolution of 18 µm and a 0.2 mm aluminum filter. Three‐dimensional reconstructions were performed using CTVol software, enabling precise visualization and analysis of the datasets. Histological analysis was performed to observe calcium oxalate crystal formation and renal injury in renal tissues.

### 2.15 Gut Microbiota Depletion and Fecal Microbiota Transplantation (FMT)

Male C57BL/6 mice (6–8 weeks old, 20–25 g) were housed under SPF conditions and free access to standard chow and water.

Donor mice preparation: Donor mice were randomly assigned to two groups (*n* = 6 per group): the Con donor group received intraperitoneal injections of an equal volume of sterile saline (vehicle control), while the Gly donor group received intraperitoneal injections of glyoxylic acid (75 mg/kg/day) dissolved in sterile saline for 14 consecutive days to induce CaOx stone formation. Fresh fecal pellets were collected from each donor group under sterile conditions at day 14, pooled by group, and suspended in sterile PBS for FMT.

Recipient mice preparation and antibiotic‐depletion experiment: Recipient mice were first treated with a broad‐spectrum antibiotic cocktail (ABX) in drinking water for 7 days to deplete the gut microbiota. The ABX cocktail consisted of ampicillin (1 g/L), vancomycin (0.5 g/L), metronidazole (1 g/L), and neomycin (1 g/L). After ABX treatment, mice were randomly divided into the following groups (*n* = 6 per group): Ctrl (normal drinking water), ABX_Sham (antibiotic‐depleted), ABX_Con (antibiotic‐depleted), and ABX_Gly (antibiotic‐depleted). Fresh fecal pellets were collected from each group at day 7 for subsequent 16S rRNA sequencing and targeted bile acid metabolomics analysis.

For FMT, separate groups of ABX‐pretreated recipient mice were subjected to glyoxylic acid‐induced CaOx stone formation (75 mg/kg/day, i.p.) for 14 consecutive days. During this period, each mouse received a daily oral gavage of 200 µL freshly prepared fecal suspension (0.2 g/mL in sterile PBS) from either Con or Gly donors. The recipient mice were assigned to three groups (*n* = 6 per group): Con+FMT_Con (Con donor microbiota, without glyoxylic acid induction), FMT_Con (Con donor microbiota, with glyoxylic acid induction), and FMT_Gly (Gly donor microbiota, with glyoxylic acid induction). All mice were maintained on the same diet and drinking water throughout the experimental period. At the end of this period, all animals were euthanized for sample collection. Fecal samples were collected from recipients for 16S rRNA sequencing and targeted bile acid metabolomics. Blood samples were collected for serum biochemical analysis. Kidney tissues were harvested for further analysis.

### 2.16 Cell Culture, Treatment, Lentiviral Transduction, Proliferation Assays, and Western Blot Assays

HK‐2 (RRID: CVCL_0302) and NRK‐52E (RRID: CVCL_0468) cell lines were obtained from the Chinese Academy of Sciences (Shanghai, China) in August 2022. They were cultured in DMEM/F12 medium for HK‐2 cells and DMEM medium for NRK‐52E. To construct a CaOx kidney stone cell model, HK‐2 and NRK‐52E cells were treated with 1 mM oxalate (cat#HY‐Y0262, MedChemExpress, USA) for 24 h according to the previous study [28]. Next, cells were treated with different concentrations of isoalloLCA (cat#HY‐B0172A, MedChemExpress, USA) for 24 h. Cell proliferation in HK‐2 and NRK‐52E cells was quantified using a Cell Counting Kit‐8 (CCK‐8) kit, with absorbance readings at 450 nm after oxalate and isoalloLCA treatment for 24 h.

For lentiviral transduction, HK‐2 and NRK‐52E renal tubular epithelial cells were seeded in 6‐well plates and cultured until they reached approximately 40% confluence. The cells were then transduced with either a lentiviral vector overexpressing PARP1 (designated as PARP1 group) or a negative control lentivirus (designated as NC group). Transduction efficiency was confirmed by western blot. Subsequently, the transduced cells were subjected to a 24 h treatment with 1 mmol/L oxalate to induce injury.

For protein analysis, whole‐cell lysates were prepared and separated by 10% SDS‐polyacrylamide gel electrophoresis (SDS‐PAGE). Proteins were then transferred onto polyvinylidene difluoride (PVDF) membranes (Millipore, USA). After blocking, membranes were incubated overnight at 4°C with a primary antibody specific for PAR and PARP1. The detailed information on the antibodies was also listed in Table S1.

### 2.17 Transmission Electron Microscope (TEM) and Immunofluorescence

HK‐2 and NRK‐52E cells were prepared for TEM using a scraping method. Cells were cultured to ≤70% confluence, medium removed, and fixed in prewarmed electron microscopy fixative (2.5% glutaraldehyde in 0.1 m buffer) at room temperature in the dark for 5 min. Cells were gently scraped in a single direction and collected into tubes, then pelleted by low‐speed centrifugation (3000 rpm, 3–5 min). After discarding supernatant, fresh fixative was added to resuspend pellets and fixation continued for 30 min at room temperature, and washed with PBS. Following graded ethanol dehydration and resin embedding, ultrathin sections (60–80 nm) were cut and stained with aqueous uranyl acetate and lead citrate. Images were acquired on a transmission electron microscope (HITACHI HT7800; nominal resolution ∼1.0 nm; Japan) at 120 kV.

Immunofluorescence was performed as previously described with minor modifications [29, 30]. Co‐staining was performed using a mIHC Fluorescence kit (Huilanbio Biological Technology, Shanghai, China) based on the tyramide signal amplification (TSA) technology according to the manufacture’s instruction. Briefly, HK‐2 and NRK‐52E cells were seeded in 24‐well plates, treated with or without oxalate and isoalloLCA for 24 h, and then fixed with 4% paraformaldehyde at room temperature for 15 min. After permeabilization and blocking in PBS containing 0.1% Triton X‐100 and 2% BSA, cells were incubated with an primary antibody of PAR, PARP1, AIF, or MIF overnight at 4 °C, followed by the appropriate fluorescent secondary antibody for 2 h at room temperature. The detailed information on the antibodies was also listed in Table S1.

### 2.18 Measurement of Intracellular Calcium (Ca^2+^) and Reactive Oxygen Species (ROS) Levels

For Intracellular Ca^2+^, HK‐2 and NRK‐52E cells were plated at 3000 cells/cm^2^ on collagen‐coated glass‐bottom fluorodishes and loaded with 2 µM Fura‐2/AM in HEPES buffer for 30 min at 37°C. Cells were superfused with control or test solutions at 37°C. Fluorescence at 340/380 nm was recorded every 2 s using an inverted fluorescence microscope (AxioObserver, Carl Zeiss) with emissions filtered at 510 nm.

ROS production was assessed using DHE staining with microfluorimetry detection. In vitro, treated HK‐2and NRK‐52E cells were incubated with 10 µm DHE for 60 min at 37°C, and ROS production was visualized using an inverted fluorescence microscope (TECAN, Switzerland). Additionally, malondialdehyde (MDA), glutathione peroxidase (GSH‐Px), and superoxide dismutase (SOD) activities in HK‐2 and NRK‐52E cells were quantified using MDA, GSH‐Px, and SOD assay kits (JianCheng Bioengineering, Nanjing, China).

### 2.19 Subcellular Fractionation

Mitochondrial and nuclear fractions were extracted using the Cell Fractionation Kit‐High Throughput (HT) (cat#ab109718, Abcam, USA), which enables sequential detergentbased separation of subcellular compartments from adherent cells without mechanical disruption. Following the manufacturer's protocol, cells were harvested and resuspended in Buffer A, followed by the addition of Detergent HT I to selectively permeabilize the plasma membrane. After centrifugation, the supernatant containing the cytosolic fraction was collected. The remaining pellet, containing intact mitochondria and nuclei, was resuspended in Detergent HT II to extract mitochondrial proteins. Following a second centrifugation, the supernatant was collected as the mitochondrial fraction, and the nuclear pellet was lysed with Detergent HT III to obtain the nuclear fraction. All fractions were subjected to western blot analysis.

### 2.20 Assessment of Mitochondrial Membrane Potential

Mitochondrial membrane potential was assessed using the Mitochondrial Membrane Potential and Apoptosis Detection Kit (cat#C1071, Beyotime, China). Following treatment, HK‐2 and NRK‐52E cells were harvested and stained with Mito‐Tracker Red CMXRos and Annexin V‐FITC according to the manufacturer's protocol. Mito‐Tracker Red fluorescence intensity reflects the maintenance of mitochondrial membrane potential, with decreased intensity indicating mitochondrial depolarization. Annexin V‐FITC staining detects phosphatidylserine externalization, an early marker of apoptosis.

### 2.21 Measurement of LDH, NAD^+^ and ATP Contents in Cells

LDH release was measured using the LDH Assay Kit (cat#C0016, Beyotime, China). After treatment, HK‐2 and NRK‐52E cells were centrifuged, and the supernatants were collected. LDH activity was determined by measuring absorbance at 490 nm according to the manufacturer's instructions. ATP levels were measured using the ATP Assay Kit (cat#S0026B, Beyotime, China). HK‐2 or NRK‐52E cells were lysed with ATP lysis buffer and centrifuged at 12 000 × g for 5 min at 4°C. ATP detection working solution was prepared by diluting the reagent 1:100. Then, 100 µL of working solution was added per well, followed by 20 µL of cell lysate. Luminescence was measured after a 2 s delay using a luminometer. ATP concentrations were calculated from a standard curve and normalized to protein content. NAD^+^ levels were measured using the NAD^+^/NADH Assay Kit (cat#S0175, Beyotime, China). HK‐2 and NRK‐52E cells were lysed in 200 µL of NAD^+^/NADH extraction buffer per 1 × 10^6^ cells, and the lysates were centrifuged at 12,000 × g for 10 min at 4°C. An aliquot of the supernatant was heated at 60°C for 30 min to selectively decompose NAD^+^, then mixed with ethanol dehydrogenase working solution and coloring reagent. After incubation, absorbance was read at 450 nm. NAD^+^ concentration was calculated from a standard curve and normalized to total protein content.

### 2.22 Exclusion of FXR Involvement in isoalloLCA‐mediated Effects

To assess whether isoalloLCA modulates FXR activity, we performed SPR to evaluate direct binding between isoalloLCA and FXR protein (cat#TMPH‐01355, TargetMol, USA). FXR was diluted to 20 µg/mL in immobilization buffer (10 mM sodium acetate, pH 4.5) and immobilized onto the sample channel (Fc2) of a CM5 sensor chip via amine coupling, reaching approximately 12 600 response units. IsoalloLCA was prepared at seven concentrations (1–100 µm) and injected over both channels at 20 µL/min, with 100 s association and 180 s dissociation. Additionally, HK‐2 and NRK‐52E cells were treated with isoalloLCA in the presence or absence of the FXR antagonist Guggulsterone (GS, cat#HY‐107738, MedChemExpress, USA) or Ursodeoxycholic acid (UDCA, cat#HY‐13771, MedChemExpress, USA). Cellular injury was assessed by cell viability assays.

### 2.23 Statistical Analysis

Statistical analysis was performed utilizing R software (version 4.3.0). For variables that followed a normal distribution, the *t‐test* was applied, whereas the *Wilcoxon test* was used for assessing variables that did not exhibit normal distribution. When multiple groups were analyzed, Analysis of Variance (*ANOVA*) was conducted. *p* <0.05 was considered statistically significant.

## 3 Results

### 3.1 Constructing an EG‐induced Rat Model of CaOx Kidney Stones

As shown in Figure 1A, a schematic diagram illustrated the establishment of CaOx kidney stone rats using 1% EG and 0.5% AC. The body weight of the CaOx rats significantly decreased (Figure 1B). Meanwhile, the urinary oxalate and Ca^2+^ levels significantly elevated in the CaOx rats, but the Mg^2+^ levels were reduced (Figure 1C). Histological analysis using H&E, VK, and AR staining showed dilation of renal tubules filled with CaOx crystals, renal tubular and glomerular atrophy, and interstitial inflammation, highlighting structural abnormalities in the kidneys of CaOx rats (Figure 1D). TUNEL assays indicated CaOx‐induced cell apoptosis in kidneys (Figure 1E). IHC analysis showed increased expression of markers associated with kidney stone formation (OPN, MCP‐1, and CD44) in the CaOx group (Figure 1F). DHE staining revealed elevated ROS levels in the CaOx group (Figure 1G). Quantitative analysis of TUNEL and DHE staining was performed (Figure 1H). Renal function was impaired (Figure 1I), and serum inflammatory factors (IL‐1β and IL‐6) were elevated using ELISA in the CaOx group (Figure 1J). Together, these results indicated the successful establishment of the CaOx rat models.

**FIGURE 1:**
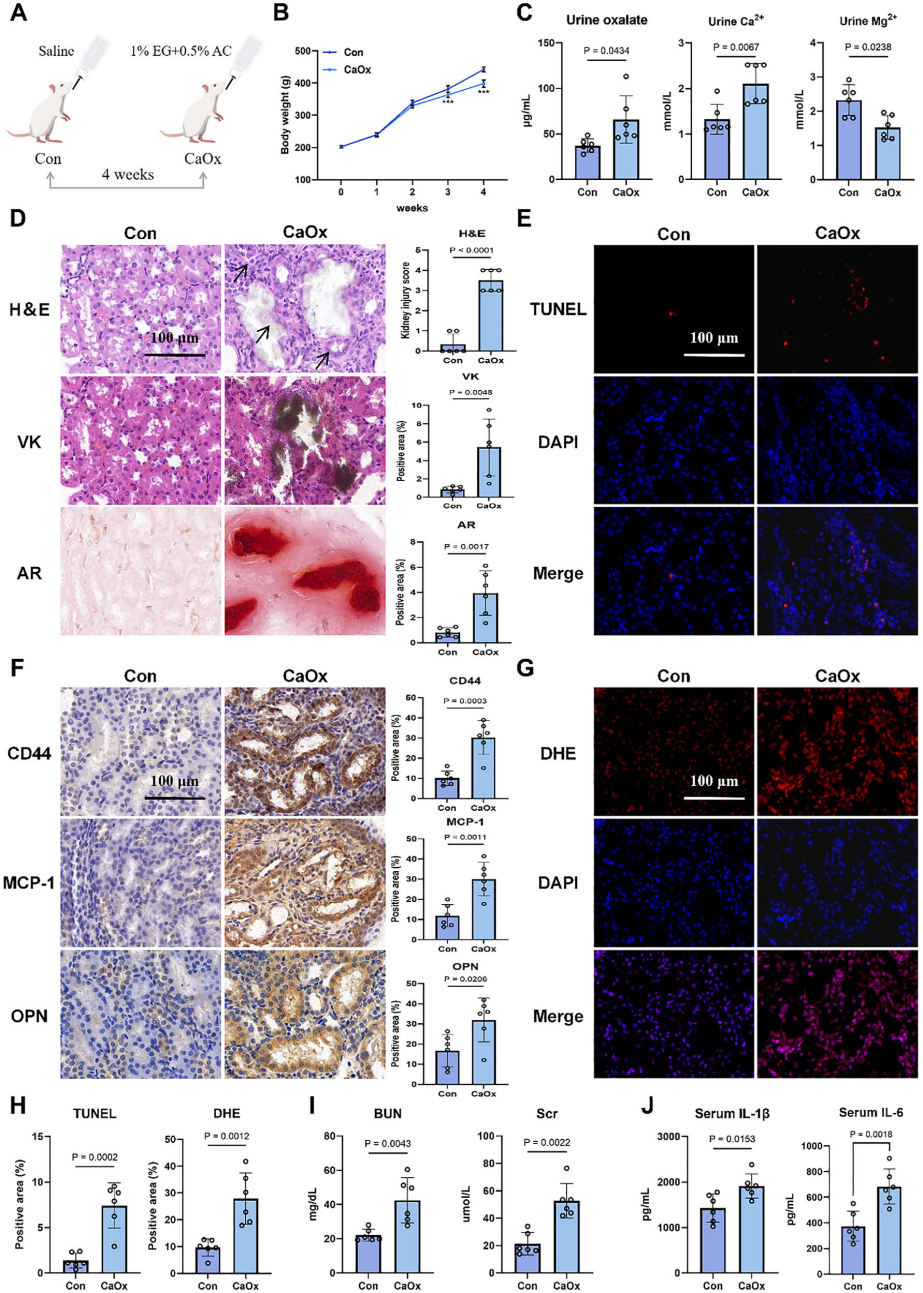
Construction and validation of CaOx kidney stone rat models. (A) The construction of CaOx kidney stone rat models using ethylene glycol (EG) and ammonium chloride (AC). (B) Body weight of rats in each group. (C) Urine oxalate, Ca^2+^, and Mg^2+^ in rats of each group. (D) Histological staining, including H&E, VK, and AR staining, shows the extent of kidney damage and CaOx crystal deposition (arrows indicate tissue damage and calcium crystals). (E) TUNEL staining of rat kidneys. (F) IHC analysis of the stone formation markers (CD44, MCP‐1, and OPN) of rat kidneys. (G) DHE staining of rat kidneys. (H) Quantitative analysis of TUNEL and DHE staining. (I) Renal function assessment in each group. (J) Serum inflammatory factors in each group measured by ELISA. (scale bar, 100 µm).

### 3.2 Targeted Metabolomics Identifies isoalloLCA as the Key Bile Acid in CaOx Nephrolithiasis

Based on our previous study on bile acids and the context of the gut‐kidney axis [18], targeted bile acid metabolomics was conducted on rat stool, serum, kidneys and urine in this study. The PCA plot highlighted differences in stool, serum, kidneys and urine bile acid profiles between the Con and the CaOx groups (Figure S1A). First, targeted bile acid metabolomics was performed on the stool. Meanwhile, the OPLS‐DA analysis indicated a distinct separation, confirming differences in bile acid profiles (Figure 2A). With critical thresholds set at *p* <0.05 and VIP>1, we identified 12 significant differential bile acids, which 3 of them were down‐regulated while 9 of them were upregulated in the CaOx group (Figure 2B). The differently expressed bile acids were displayed in descending order based on their VIP scores (Figure 2C). Subsequently, targeted bile acid metabolomics was performed on the serum of rats. The OPLS‐DA analysis indicated a distinct separation, confirming differences in bile acid profiles (Figure 2D). We identified 19 significant differential bile acids, which 17 of them were down‐regulated while 2 of them were upregulated in the CaOx group (Figure 2E). The differently expressed bile acids were displayed in descending order based on their VIP scores (Figure 2F).

**FIGURE 2:**
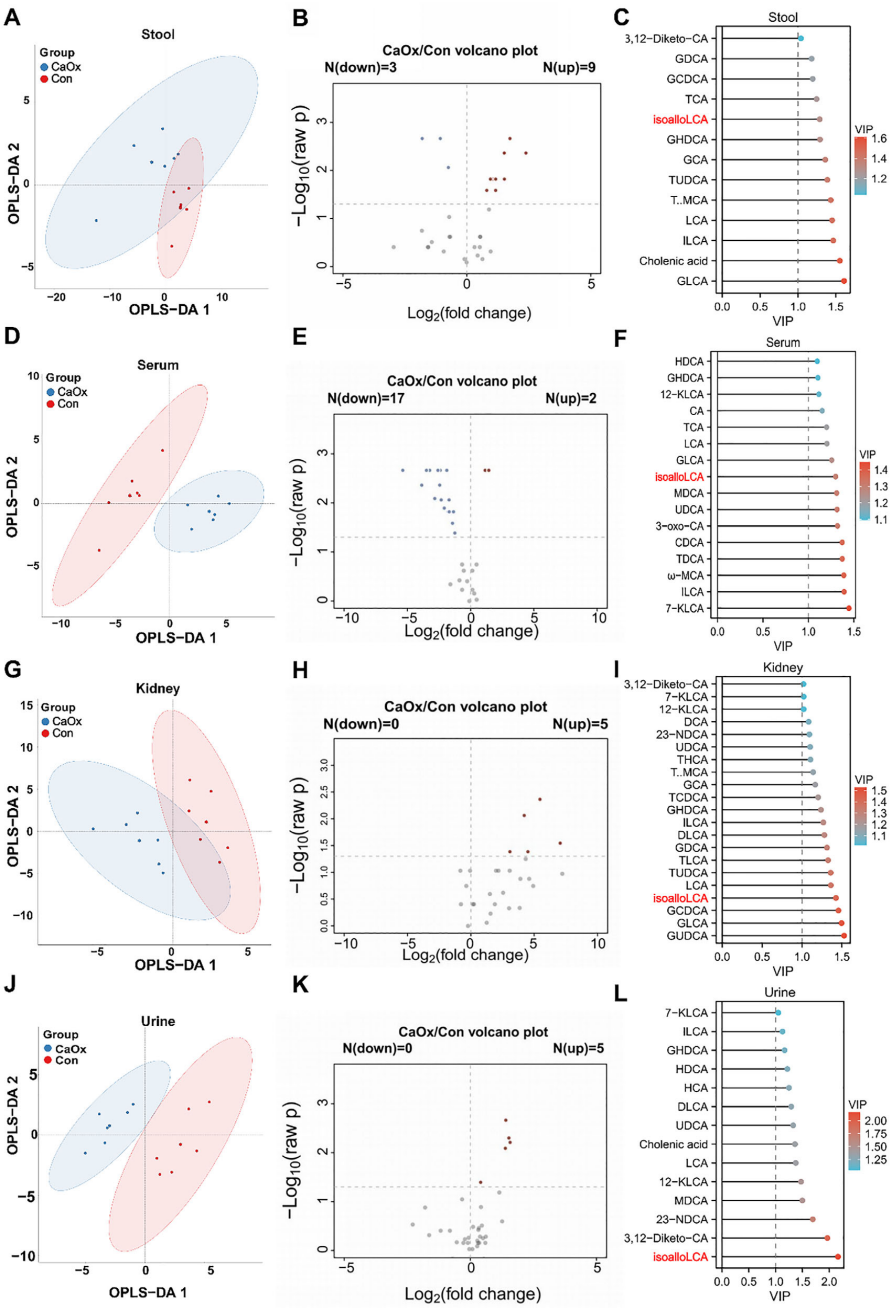
Targeted bile acid metabolomics of rat stool, serum, kidneys and urine. (A‐C) The OPLS‐DA score (A), volcano plot (B), and VIP score plot (C) of differential bile acids in rat stool between Con and CaOx groups. (D‐F) The OPLS‐DA score (D), volcano plot (E), and VIP score plot (F) of differential bile acids in rat serum between Con and CaOx groups. (G‐I) The OPLS‐DA score (G), volcano plot (H), and VIP score plot (I) of differential bile acids in rat kidneys between Con and CaOx groups. (J‐L) The OPLS‐DA score (J), volcano plot (K), and VIP score plot (L) of differential bile acids in rat urine between Con and CaOx groups.

Following, targeted bile acid metabolomics was performed on the kidneys and urine of rats. The OPLS‐DA analysis indicated a distinct separation, confirming differences in bile acid profiles of rat kidneys (Figure 2G) and urine (Figure 2J). We identified 5 significant differential bile acids in kidneys (Figure 2H) and in urine (Figure 2K). The differently expressed bile acids in kidneys (Figure 2I) and in urine (Figure 2L) were displayed in descending order based on their VIP scores.

We identified isoalloLCA as the crucial differential bile acid by analyzing the overlap among stool, serum, kidneys and urine profiles with Veen diagram (Figure 3A), suggesting that isoalloLCA was the key bile acid in the development of CaOx kidney stones via the gut‐kidney axis. As shown in Figure 3B, isoalloLCA was significantly upregulated in the CaOx group based on the targeted bile acid metabolomics profiles of rat stool, serum, kidneys, and urine.

**FIGURE 3:**
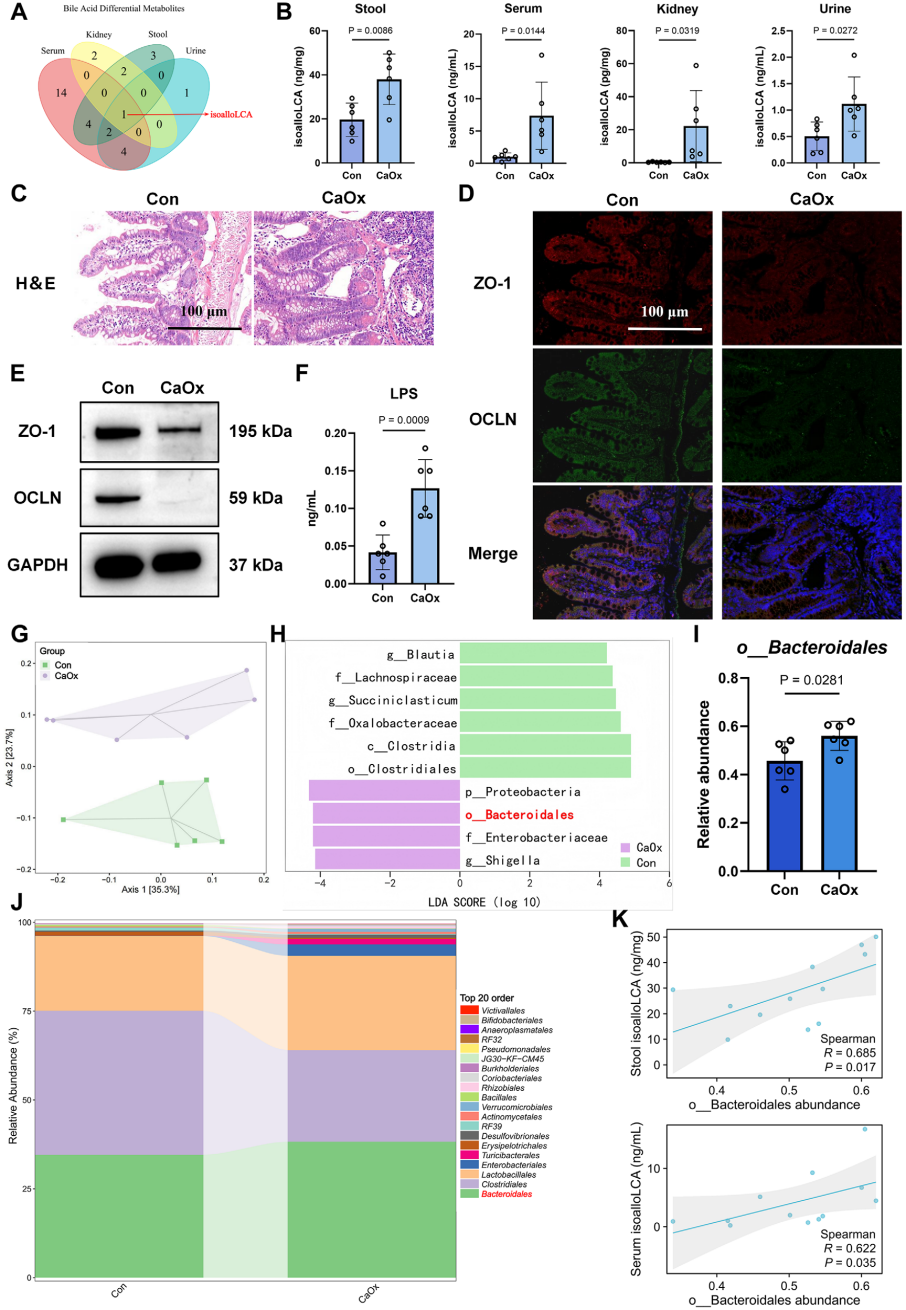
Altered bile acid profile, intestinal barrier damage, and gut microbiota dysbiosis in CaOx rats. (A) Venn diagram of the intersection of stool, serum, renal, and urine differential bile acids. (B) The levels of isoalloLCA in stool, serum, kidneys, and urine between Con and CaOx groups using targeted metabolomics. (C) Representative H&E staining of the ileal epithelial tissue. (D‐E) The immunofluorescence staining (D) and western blot analysis (E) of ZO‐1 and OCLN in ileal tissues (scale bar, 100 µm). (F) Serum LPS levels in each group. (G‐H) The PCoA (G) and LEfse (H) analysis analyzed by 16S sequencing. (I) Relative abundance of *o_Bacteroidales* between groups. (J) Species annotation at order levels. (K) Spearman correlation analysis of *o_Bacteroidales* with isoalloLCA in stool and serum.

### 3.3 Alteration of the Intestinal Permeability and Gut Microbiota in CaOx Rats

Increased intestinal inflammation and compromised barrier function are implicated in kidney diseases through the gut‐kidney axis [31]. Therefore, this study assessed the intestinal barrier integrity and gut microbiota in the rats. H&E staining revealed that the ileal epithelium in the Con group maintained intact architectural integrity with clearly defined layers; however, the CaOx group exhibited ileum epithelial swelling and congestion with notable inflammatory cell infiltration (Figure 3C). Additionally, there was a marked decrease in the expression of tight junction proteins, ZO‐1 and OCLN, in the ileum of the CaOx group, as shown by dual immunofluorescence staining (Figure 3D) and western blot analysis (Figure 3E). Meanwhile, the LPS levels in serum were significantly elevated in the CaOx group (Figure 3F).

When comparing microbial diversity, the PCoA analysis of beta diversity indicated distinct microbial community clustering between the two groups (Figure 3G). LEfSe analysis identified *c_Clostridia* and *o_Clostridiales* as the dominant taxa in the Con group and *o_Bacteroidales* in the CaOx group (Figure 3H). The relative abundance of *o_Bacteroidales* was significantly higher in the CaOx group (Figure 3I). Notably, *o_Bacteroidales* is a primary microbial reservoir for isoalloLCA, including *Parabacteroides merdae, Bacteroides dorei*, and *Bacteroides vulgatus* [32, 33]. Species annotation confirmed a higher relative abundance of the *p_Bacteroidetes, c_Bacteroidia* (Figure S1B), and *o_Bacteroidales* (Figure 3J) in the CaOx group. The CaOx group showed a significant decrease in alpha diversity as determined by 16S rRNA sequencing (Figure S1C). Scatterplots with Spearman correlation analysis revealed a positive correlation between the abundance of *o_Bacteroidales* and isoalloLCA levels in both stool and serum (Figure 3K), as well as in kidney and urine (Figure S1D).

### 3.4 Dysbiosis of isoalloLCA Is Associated With Gut Microbiota in CaOx Stone Individuals

As described above, we collected 20 fecal samples from both the HC group and the CaOx group for metagenomic sequencing and targeted bile acid metabolomics (Table S2). The gut microbiota of CaOx stone formers exhibited significant dysbiosis, characterized by reduced α‑diversity compared with healthy controls (Figure 4A). The relative abundance of the genus *Faecalibacterium* and the species *Faecalibacterium prausnitzii* (*F. prausnitzii*) was significantly lower, while that of *Ruminococcus gnavus* (*R. gnavus*) was markedly higher, in individuals with CaOx renal stones (Figure 4B–D). For targeted bile acid metabolomics, the scatter diagram showed that isoalloLCA levels were significantly elevated in the CaOx group (*P* = 0.0077) and the ROC curve demonstrated the discriminatory power of isoalloLCA levels between the HC group and CaOx group (AUC = 0.80, Figure 4E). Scatterplots with Spearman correlation analysis revealed revealed that isoalloLCA levels were negatively correlated with the abundance of *g_Faecalibacterium* and *F. prausnitzii*, but positively correlated with the abundance of *R. gnavus* (Figure 4F). IsoalloLCA is generated from chenodeoxycholic acid (CDCA) through sequential 7α‑dehydroxylation (Bai operon), 3α‑hydroxysteroid dehydrogenase (3α‑HSDH), 5β‑reductase, 5α‑reductase, and 3β‑hydroxysteroid dehydrogenase (3β‑HSDH) [33] (Figure 4G). In the CaOx group, the expression of BaiB, BaiH, 3α‑HSDH, and 5α‑reductase was markedly enriched, indicating the production of isoalloLCA (Figure 4H).

**FIGURE 4:**
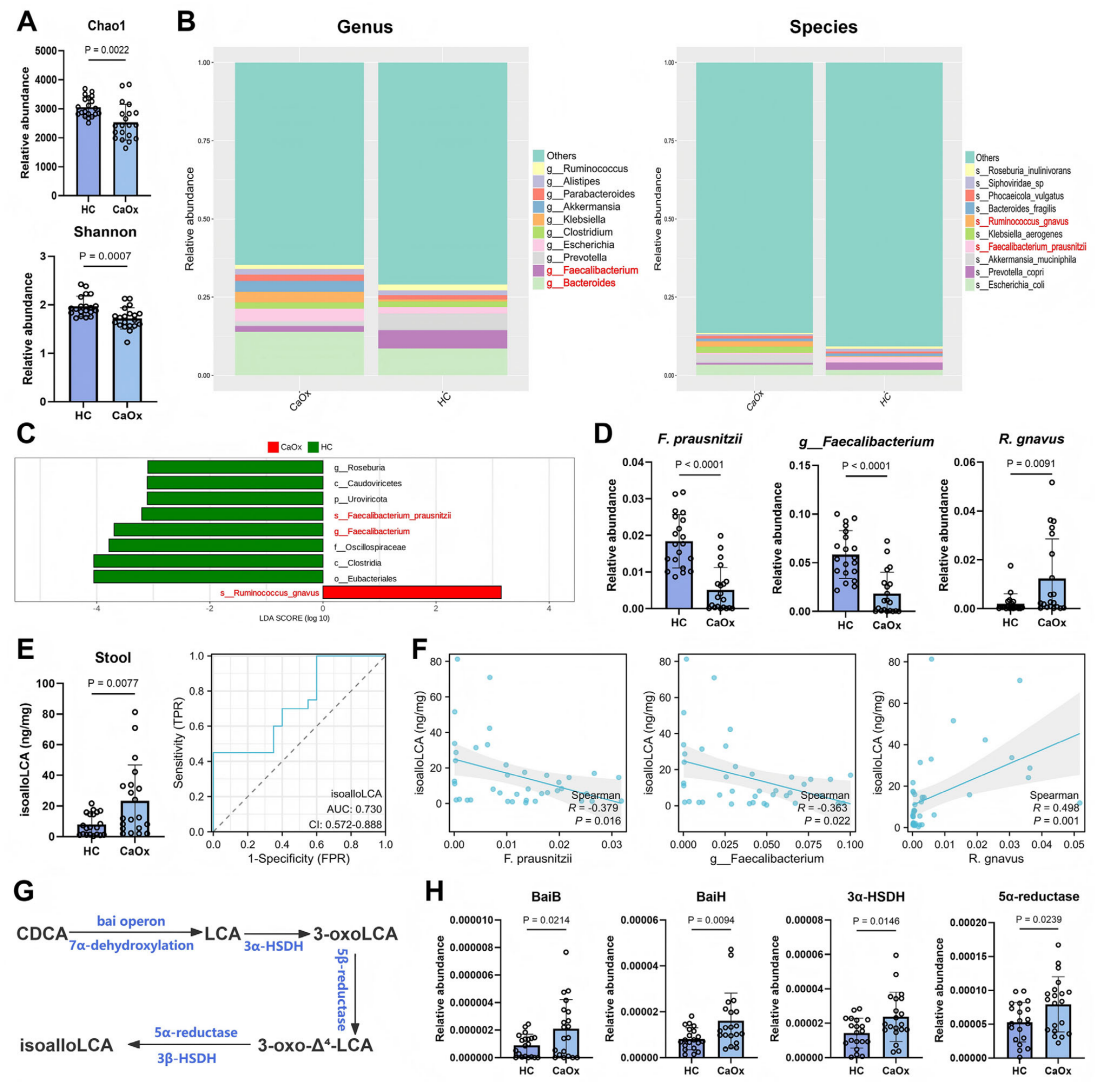
Dysbiosis of isoalloLCA in CaOx stone individuals is associated with gut microbiota. (A) Reduced α‑diversity in the gut microbiota of CaOx stone formers (*n* = 20) compared with healthy controls (*n* = 20). (B) Relative abundance of genus and species in feces of healthy controls and CaOx stone formers. (C) LDA effect size analysis between groups. (D) Relative abundance of *g_Faecalibacterium*, *F. prausnitzii*, and *R. gnavus* between groups. (E) Elevated isoalloLCA levels in the CaOx group and the receiver operating characteristic curve. (F) Spearman correlation analysis of isoalloLCA with *g_Faecalibacterium*, *F. prausnitzii*, and *R. gnavus* (*n* = 40). (G) Schematic of isoalloLCA biosynthesis from chenodeoxycholic acid (CDCA). (H) Enriched expression of genes‐related isoalloLCA biosynthesis in the CaOx group. (HC: healthy control, CaOx: calcium oxalate).

### 3.5 CaOx Rat Serum and Kidney Proteomics Analysis

Proteomic analysis was carried out on serum and renal tissues of rats. In the serum, the volcano plot was created to visualize the distribution of differentially expressed proteins (DEPs) using a threshold of *P* < 0.05 and |log_2_FC| > 1, revealing that 102 proteins were upregulated and 142 proteins were downregulated in the CaOx group (Figure 5A, Table S3). The heatmap of the DEPs was displayed in Figure S2A. The subcellular localization of DEPs in serum was shown in Figure 5B. KEGG pathway analysis indicated significant enrichment in apoptosis, ferroptosis, cell adhesion molecules, etc. (Figure 5C). GO analysis suggested that these DEPs predominantly participate in tight junction, cell‐substrate adhesion, and other functions (Figure S2B). Further GSEA analysis showed a significant enrichment in necroptosis, cell cycle and else (Figure S2C), which are vitally associated with kidney stone development.

**FIGURE 5:**
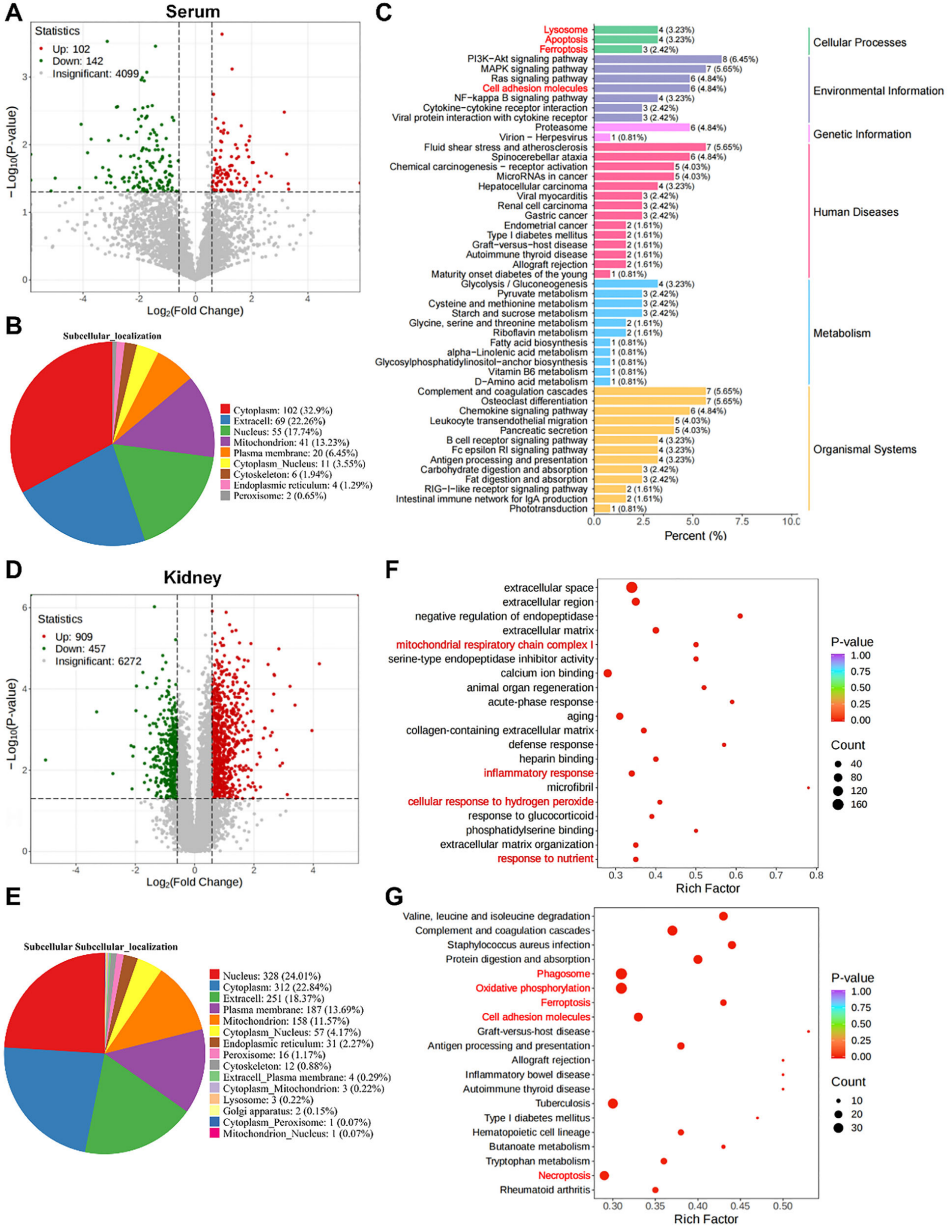
Proteomics analysis of rat serum and kidneys. (A‐B) The volcano plot (A) and subcellular localization (B) of differentially expressed proteins (DEPs) in serum between Con and CaOx groups. (C) KEGG enrichment analysis of DEPs in serum. (D‐E) The volcano plot (D) and subcellular localization (E) of DEPs in kidneys. (F‐G) GO (F) and KEGG (G) enrichment analysis of DEPs in kidneys.

In the kidneys, the volcano plot revealed that 909 proteins were upregulated and 457 proteins were downregulated in the CaOx group (Figure 5D, Table S4). The subcellular localization of DEPs in serum was shown in Figure 5E. The heatmap of the DEPs was displayed in Figure S2D. The PCA plot highlighted differences in kidney proteins between the Con and the CaOx groups (Figure S2E). GO analysis suggested that these DEPs predominantly participate in the inflammatory response, extracellular matrix, calcium ion binding, cellular response to hydrogen peroxide, response to nutrient, etc. (Figure 5F). KEGG pathway analysis indicated significant enrichment in the pathways, including oxidative phosphorylation, ferroptosis, cell adhesion molecules, tryptophan metabolism, necroptosis, etc. (Figure 5G). Further GSEA analysis showed a significant enrichment in cholesterol metabolism, apoptosis, and else (Figure S2F), suggesting the potential role of bile acids in CaOx kidney stones.

### 3.6 Spatial Metabolomics and Network Pharmacology Identifying isoalloLCA as the Key Bile Acid and Its Target PARP1

Using spatial metabolomics multimodal analysis of kidneys, we detected isoalloLCA accumulation specifically in kidney regions associated with tubular injury (Figure 6A). Next, we utilized network pharmacology to identify the target of isoalloLCA. Analysis utilizing the CTD database indicated 106 genes associated with CaOx kidney stones (Figure 6B, Table S5), while the PharmMapper database revealed 292 potential targets for isoalloLCA using network pharmacology approaches (Figure 6C, Table S6). By intersecting these datasets with DEPs in rat serum and kidneys, we identified PARP1 as the critical target of isoalloLCA in CaOx kidney stones (Figure 6D). The PARP1 protein expression was elevated in the serum and kidneys of the CaOx rats based on the proteomics analysis (Figure 6E). Meanwhile, western blot (Figure 6F) and IHC (Figure 6G) analysis revealed that the PARP1 protein expression was elevated in the kidneys of the CaOx rats.

**FIGURE 6:**
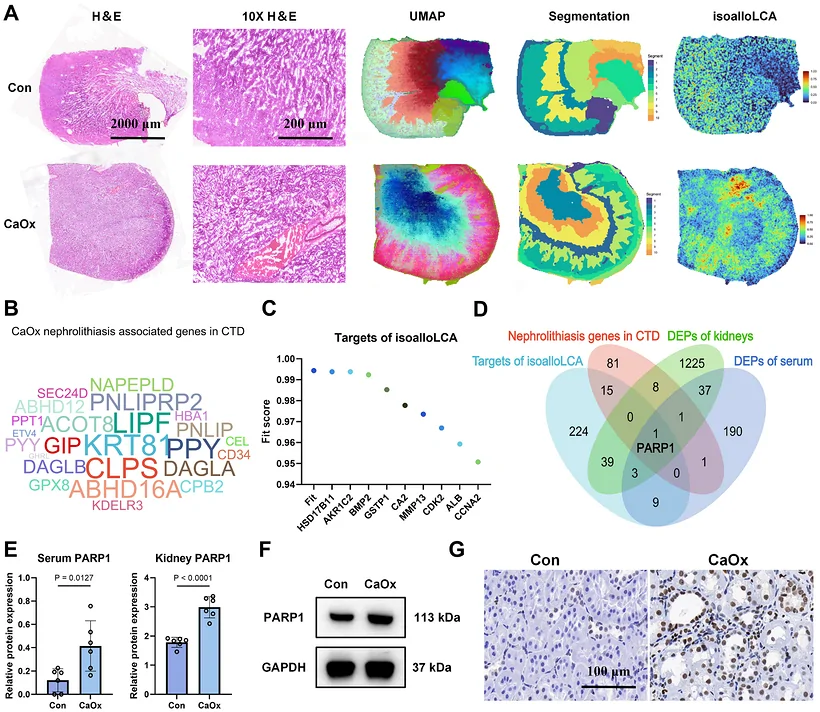
Spatial metabolomics and network pharmacology identify isoalloLCA as the key bile acid and its target PARP1. (A) Spatial metabolomics reveals colocalization of isoalloLCA with tubular injury in CaOx group. (B) Word cloud diagram of the CaOx nephrolithiasis associated genes in the Comparative Toxicogenomics Database (CTD). (C) Target proteins of isoalloLCA in the PharmMapper database. (D) Venn Diagram of the intersection of isoalloLCA targets, differentially expressed proteins in serum and kidneys, and CaOx nephrolithiasis associated genes in CTD, identifying the target protein of isoalloLCA, PARP1. (E) The protein expression of PARP1 in serum and kidneys between Con and CaOx groups based on proteomics. (F‐G) The protein expression of PARP1 in rat kidneys using western blot (F) and IHC analysis (G). (scale bar, 100 µm).

### 3.7 IsoalloLCA Exhibits a Potent Binding Affinity for PARP1 Protein

To further explore the interaction between isoalloLCA and the PARP1 protein, we conducted a series of biophysical assays, including SPR, MST, CETSA, MD simulation, and molecular docking. SPR analysis demonstrated a direct interaction between isoalloLCA and the PARP1 protein, with an equilibrium dissociation constant (Kd) of 4.31 µm (Figure 7A,B). Additionally, MST results yielded a Kd value of 8.0 µm (Figure 7C), and the full binding curves are provided in Figure S3A. Additionally, molecular docking studies demonstrated interactions between isoalloLCA and human PARP1 protein with a hydrogen bond energy of −9.4 kcal/mol (Figure 7D) and rat PARP1 protein with a hydrogen bond energy of −8.8 kcal/mol (Figure 7E). Furthermore, to evaluate the stability and conformational behavior of the protein‐ligand (PARP1‐isoalloLCA) complex during the simulation, root mean square deviation (RMSD), root mean square fluctuation (RMSF), radius of gyration (Rg), solvent‐accessible surface area (SASA), and hydrogen bonding (Hbond) were analyzed (Table S7). The RMSD of the C‐alpha atoms indicated that the protein–ligand complex achieved stability during the first 2 ns of the simulation, with no significant fluctuations observed over the 100 ns trajectory (Figure 7F). The RMSF analysis revealed lower flexibility for residues before position 200, while residues beyond position 200 exhibited higher flexibility (Figure 7G). Hydrogen bond analysis confirmed stable intramolecular hydrogen bonding, indicating no conformational unfolding during the simulation (Figure 7H). The stable Rg (48.6–48.9 Å, Figure S3B) and consistent SASA (Figure S3C) throughout the simulation confirmed that no unfolding or major conformational changes occurred. CETSA validated the thermodynamic stability of isoalloLCA binding to PAPR1, showing enhanced stability of PAPR1 (54°C–67°C) in HK‐2 and NRK‐52E cells (Figure 7I). These findings collectively confirm the interaction between isoalloLCA and PARP1, providing quantitative insights into their binding affinity and thermal stability changes upon interaction.

**FIGURE 7:**
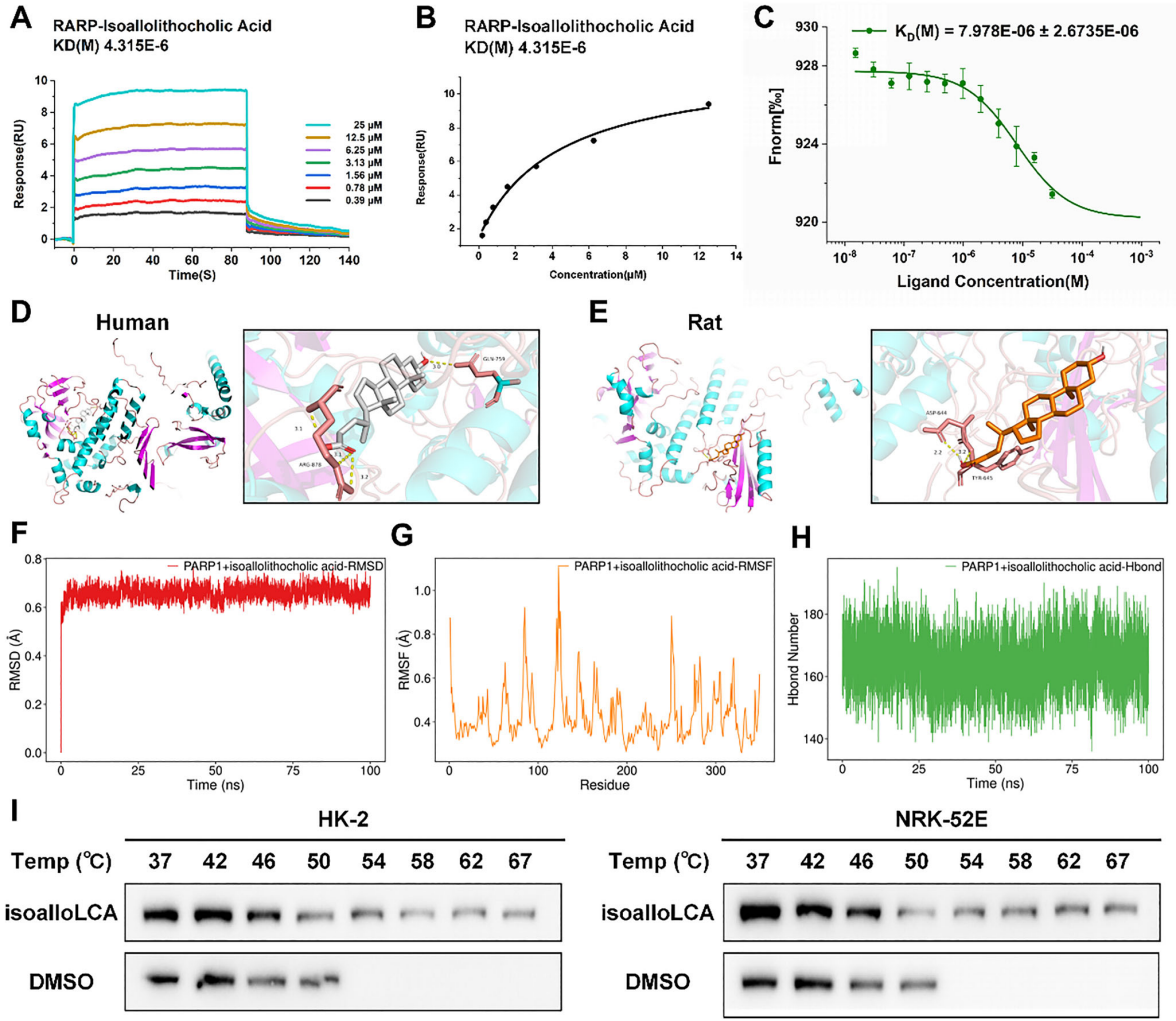
isoalloLCA exhibits a potent binding affinity for PARP1. (A‐B) SPR analysis of the binding affinity of isoalloLCA to PARP1 protein. The Kd (mol/L) value between isoalloLCA and PARP1 was 4.31 µm. (C) Microscale thermophoresis (MST) analysis of isoalloLCA binding to PARP1. The Kd (mol/L) value between isoalloLCA and PARP1 was 8.0 µm. (D‐E) Molecular docking of isoalloLCA with human (D) and rat (E) PARP1 protein. (F) RMSD graph of isoalloLCA with PARP1. (G) RMSF graph of isoalloLCA with PARP1. (H) H‐bond contacts of isoalloLCA with PARP1. (I) CETSA assessed isoalloLCA binding to PARP1 in HK‐2 and NRK‐52E cells by measuring temperature‐dependent PARP1 protein stability.

### 3.8 IsoalloLCA Promotes Kidney Stone Formation via PARP1 in CaOx Rats and in Glyoxylic Acid‐induced CaOx Mice

To determine if isoalloLCA‐mediated stone‐promoting effect was through PARP1, we knocked down PARP1 in rat kidneys using AAV carrying shRNA targeting PARP1 coding sequence, and fed rat models with feed containing 1% isoalloLCA (Figure 8A). The knockdown of PARP1 in the rat kidney was confirmed by western blot (Figure 8B). As shown in Figure 8C, PARP1 knockdown by AAV‐shPARP1 blocked the elevation in BUN and Scr levels caused by isoalloLCA treatment in the CaOx rats. The knockdown of PARP1 in the kidney was further confirmed by immunofluorescence staining (Figure 8D). As determined by H&E and VK staining, PARP1 knockdown inhibited the aggravation of CaOx deposition and tubular injury by isoalloLCA treatment, while TUNEL staining indicated that PARP1 knockdown blocked the isoalloLCA‐mediated cell apoptosis in the CaOx rats (Figure 8D). Immunofluorescence staining revealed that PARP1 expression in intestinal tissues closely resembled that observed in renal tissues, with comparable localization patterns and fluorescence intensity (Figure S4). This parallel expression profile suggests a potential functional linkage along the gut‑kidney axis.

**FIGURE 8:**
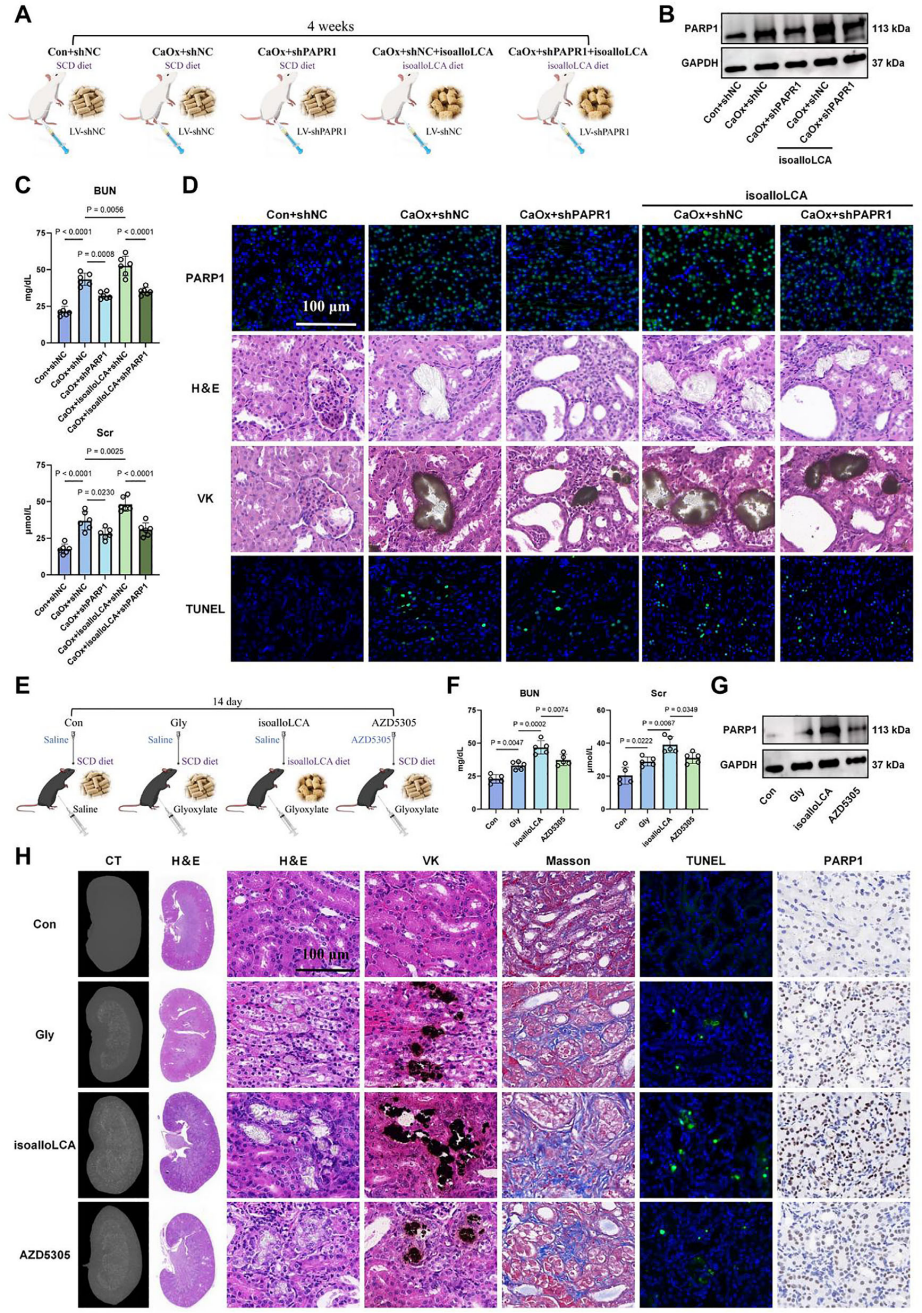
isoalloLCA promotes kidney stone formation via targeting PARP1 in CaOx rats and in glyoxylic acid‐induced CaOx mice. (A) A schematic diagram of the isoalloLCA treatment and PAPR1 knockdown by AAV in CaOx rats. (B) Detection of PARP1 protein expression in CaOx rats by western blot. (C) PAPR1 knockdown blocked the isoalloLCA‐mediated aggravation of renal function in CaOx rats (BUN: blood urea nitrogen, Scr: serum creatinine). (D) Representative images of PARP1 IHC immunofluorescence, H&E, VK, and TUNEL staining in CaOx rats. (E) A schematic diagram of the isoalloLCA treatment and PAPR1 inhibitor (AZD5305) on glyoxylic acid‐induced CaOx mice. (F) PAPR1 inhibitor blocked the isoalloLCA‐mediated aggravation of renal function in CaOx mice. (G) Detection of PARP1 protein expression in CaOx mice by western blot. (H) Representative images of micro‐CT, H&E, VK, Masson, TUNEL, and PARP1 IHC staining in CaOx mice. (Data are presented as mean ± SD. *p*‐values were determined by one‐way ANOVA. scale bar, 100 µm).

Mice were administered intraperitoneal injections of glyoxylic acid (75 mg/kg) daily for 14 consecutive days to establish a CaOx nephrolithiasis mouse model (named Gly group). To determine if the isoalloLCA‐mediated kidney stone formation was through PAPR1, we fed mice models with feed containing 1% isoalloLCA (named isoalloLCA group), and used the PARP1 inhibitor AZD5305 intraperitoneally injecting to inhibit PARP1 expression (named AZD5305 group) (Figure 8E). isoalloLCA promoted the body weight loss of the Gly mice, while PARP1 inhibition by AZD5305 rescued it (Figure S5A). Meanwhile, PARP1 inhibition by AZD5305 blocked the increase in the BUN, Scr levels (Figure 8F), and inflammatory factors (IL‐1β and IL‐6) by isoalloLCA treatment in the Gly group (Figure S5B). The knockdown of PARP1 in the mice kidney was confirmed by western blot (Figure 8G). Micro‐CT exhibited obvious renal calcium crystal deposition in the Gly mice, which was markedly aggravated in the isoalloLCA group, but was reduced in the AZD5305 group. As determined by H&E and VK staining, PARP1 inhibition by AZD5305 relieved the aggravation of CaOx deposition and tubular injury by isoalloLCA treatment (Figure 8H). The full‐field Von Kossa staining images of each group of mice were shown in Figure S5C. Masson staining showed PARP1 inhibition blocked the isoalloLCA‐mediated renal fibrosis, while TUNEL staining indicated that PARP1 inhibition blocked the isoalloLCA‐mediated cell apoptosis in the Gly mice (Figure 8H). Also, AZD5305 blocked the upregulated positive staining of PARP1 by isoalloLCA treatment using IHC analysis (Figure 8H). These findings support a mechanistic role of isoalloLCA in promoting kidney stone formation via targeting PAPR1.

### 3.9 PARP1 Inhibition Attenuates Crystal Deposition and Renal Injury in Glyoxylic Acid‐induced CaOx Mice

To evaluate the role of PARP1 CaOx nephrolithiasis, mice were divided into four groups (Figure S6A): the Con group (saline i.p. + saline gavage), the AZD5305 group (saline i.p. + AZD5305 gavage), the Gly group (glyoxylic acid i.p. + saline gavage), and the Gly + AZD5305 group (glyoxylic acid i.p. + AZD5305 gavage). Body weight changes during the treatment period were monitored, and no significant differences were observed between the Con and the AZD5305 group, but AZD5305 treatment significantly ameliorated the weight loss in glyoxylic acid‐induced mice (Figure S6B). Representative H&E and VK staining of kidney sections showed that AZD5305 treatment significantly reduced crystal deposition and tubular injury in glyoxylic acid‐induced mice (Figure S6C), as confirmed by quantitative analysis (Figure S6D). Renal function assessment revealed that BUN and Scr levels were markedly elevated in the Gly group, whereas co‐treatment with AZD5305 significantly attenuated these changes (Figure S6E). Additionally, serum levels of inflammatory cytokines IL‐1β and IL‐6, were increased in the Gly group but were significantly reduced by AZD5305 administration (Figure S6F). These results collectively indicate that PARP1 inhibition alleviates crystal deposition, renal dysfunction, and inflammation in glyoxylic acid‐induced CaOx mice.

### 3.10 Gut Microbiota Contributes to isoalloLCA‐Mediated CaOx Stone Formation in CaOx Mice

The schematic diagram of the ABX and FMT experiment is shown in Figure 9A. 16S rRNA sequencing revealed that the gut microbiota of the ABX_Con and ABX_Gly groups exhibited severe depletion and markedly reduced α‐diversity, confirming successful antibiotic clearance (Figure 9B). The PCoA analysis revealed significant differences in gut microbiota composition among three groups (Figure 9C). At the phylum level, antibiotic treatment shifted the dominant taxon from *p_Bacteroidota* and *p_Bacillota* in controls to *p_Pseudomonadota* (Figure 9D). LEfSe analysis further confirmed this result (Figure 9E). A significant decrease in isoalloLCA levels was observed following microbiota depletion in the ABX_Con and ABX_Gly groups (Figure 9F).

**FIGURE 9:**
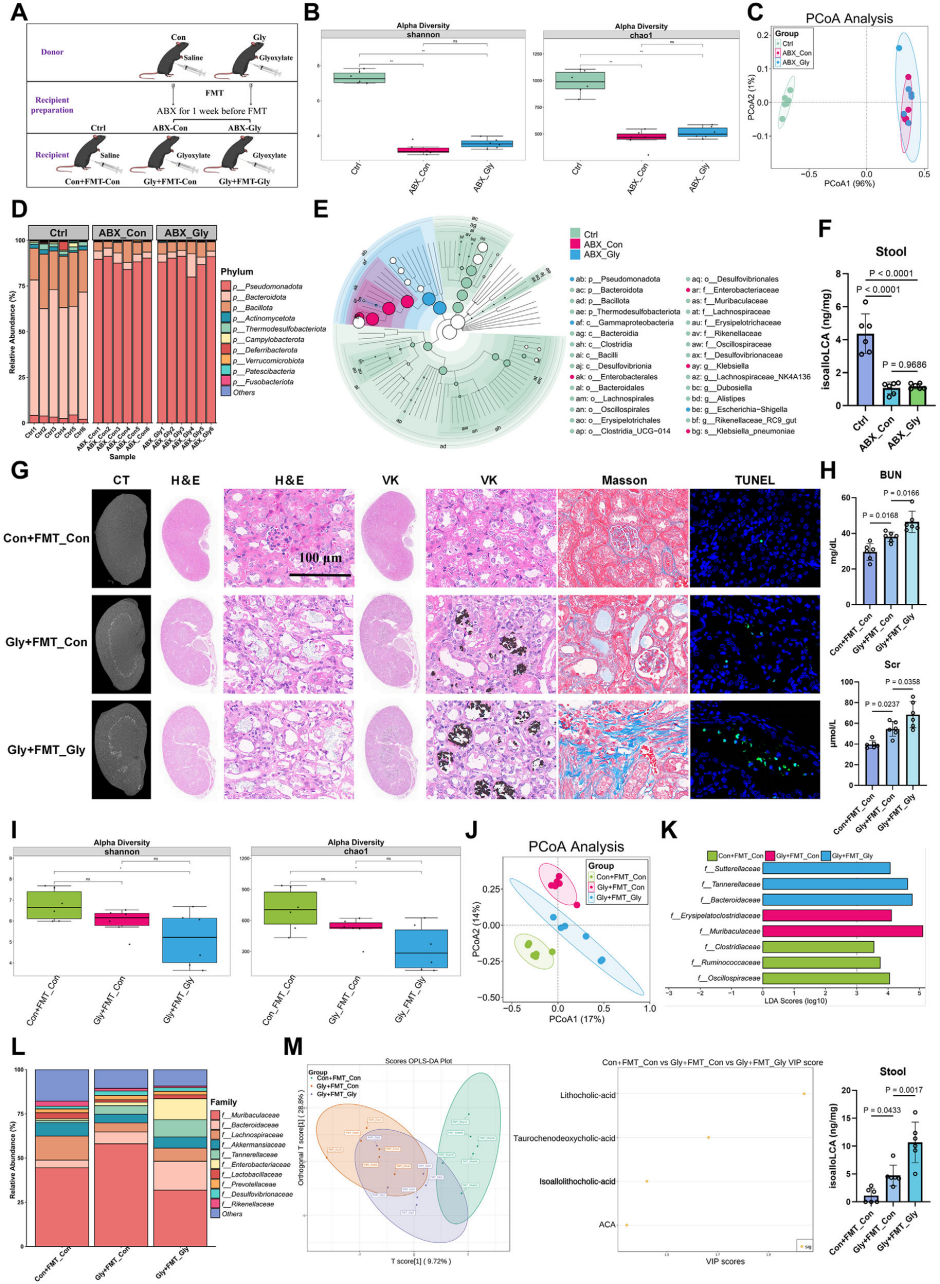
Fecal microbiota transplantation (FMT) establishes a causal role of gut microbiota in isoalloLCA‐mediated CaOx nephrolithiasis. (A) The schematic diagram of the FMT experiment. (B) Alpha diversity showing restored diversity in ABX_Con and ABX_Gly groups. (C‐E) The PCoA plot (C), phylum‐level relative abundance (D), and the LEfSe analysis (E) among the Ctrl, ABX_Con and ABX_Gly groups. (F) The isoalloLCA levels in ABX groups using metabolomics. (G) Representative images of micro‐CT, H&E, VK, Masson, and TUNEL staining in FMT mice. (H) Renal function in FMT mice. (I‐L) Alpha diversity (I), PCoA plot (J), LEfSe analysis (K), and family‐level relative abundance (L) among FMT groups. (M) The isoalloLCA levels in FMT groups using metabolomics. (Data are presented as mean ± SD. *p*‐values were determined by one‐way ANOVA. scale bar, 100 µm).

Furthermore, we carried out FMT experiments. The Con+FMT_Con group showed no renal phenotype, ruling out any effect of FMT, whereas the Gly+FMT_Con group exhibited CaOx crystal deposition and tubular injury, and these injuries were markedly attenuated in the Gly+FMT_Gly group, indicating a protective effect of control microbiota transplantation (Figure 9G). Renal function assessment revealed that BUN and Scr levels showed the same pattern (Figure 9H). 16S rRNA sequencing revealed that FMT restored the gut microbiota in ABX‐pretreated mice (Figure 9I). The PCoA analysis revealed significant differences in gut microbiota composition among three groups (Figure 9J). The LEfSe analysis highlighted *f_Bacteroidaceae* as the key discriminant families in the Gly+FMT_Gly group, with *f_Ruminococcaceae* and *f_Oscillospiraceae* in the Con+FMT_Con group (Figure 9K), whereas the family‐level taxonomic profiles confirmed this result (Figure 9L). Consistently, isoalloLCA levels kidney were markedly elevated in Gly+FMT_Gly mice, whereas they remained low in Con+FMT_Con and Gly+FMT_Con recipients (Figure 9M), indicating that the stone‐forming microbiota transferred to recipients elevated isoalloLCA.

### 3.11 PARP1‐Induced Parthanatos Promoted Cell Injury in Renal Tubular Epithelial Cells

PARP1 overexpression was confirmed by western bolt following lentiviral transduction (Figure S7A). Functionally, PARP1 reduced cell viability in renal tubular epithelial cells (Figure S7B). Immunofluorescence demonstrated nuclear‐to‐cytoplasmic translocation and co‐localization of AIF (green) with PARP1 (red) in HK‐2 (Figure S7C) and NRK‐52E (Figure S7D) cells after lentiviral transduction. Moreover, PARP1 significantly exacerbated oxalate‐induced injury in renal tubular epithelial cells (Figure S7E), indicating a causal role of PARP1‐driven parthanatos in tubular cell damage.

### 3.12 IsoalloLCA Promotes PARP1‐Mediated Parthanatos in Renal Tubular Epithelial Cells

We then assessed the effects of isoalloLCA on renal tubular epithelial cells by exposing HK‐2 and NRK‐52E cells to varying concentrations (0, 50, 100, 200, and 400 µm) of isoalloLCA. The CCK‐8 assay indicated that a concentration of 200 µm isoalloLCA significantly impaired cell viability (Figure S8A), which was the concentration selected for further studies. Co‐treatment with isoalloLCA and oxalate exacerbated the cellular damage compared to the oxalate treatment alone, while PARP1 inhibition by AZD5305 inhibited the aggravation of cell injury by isoalloLCA treatment (*P*<0.05, Figure 10A). The western blot results demonstrated that the PARP1 protein level was increased in the isoalloLCA group, but was reduced in the AZD5305 group (Figure 10B). The results of calcium flow assays showed that AZD5305 inhibited isoalloLCA‐mediated calcium flow in HK‐2 and NRK‐52E cells (Figure 10C). Following isoalloLCA exposure, a marked increase in PAR polymer levels was observed by western blot (Figure 10D) and immunofluorescence analysis (Figure 10E), indicating PARP1 hyperactivation, which was reversed by the PARP1 inhibitor AZD5305. Meanwhile, in animal models, following isoalloLCA exposure, a marked increase in PAR polymer and H2AX levels was observed by immunofluorescence staining in CaOx mouse (Figure S8B) and rats (Figure S8C), which was reversed by the PARP1 inhibitor AZD5305.

**FIGURE 10:**
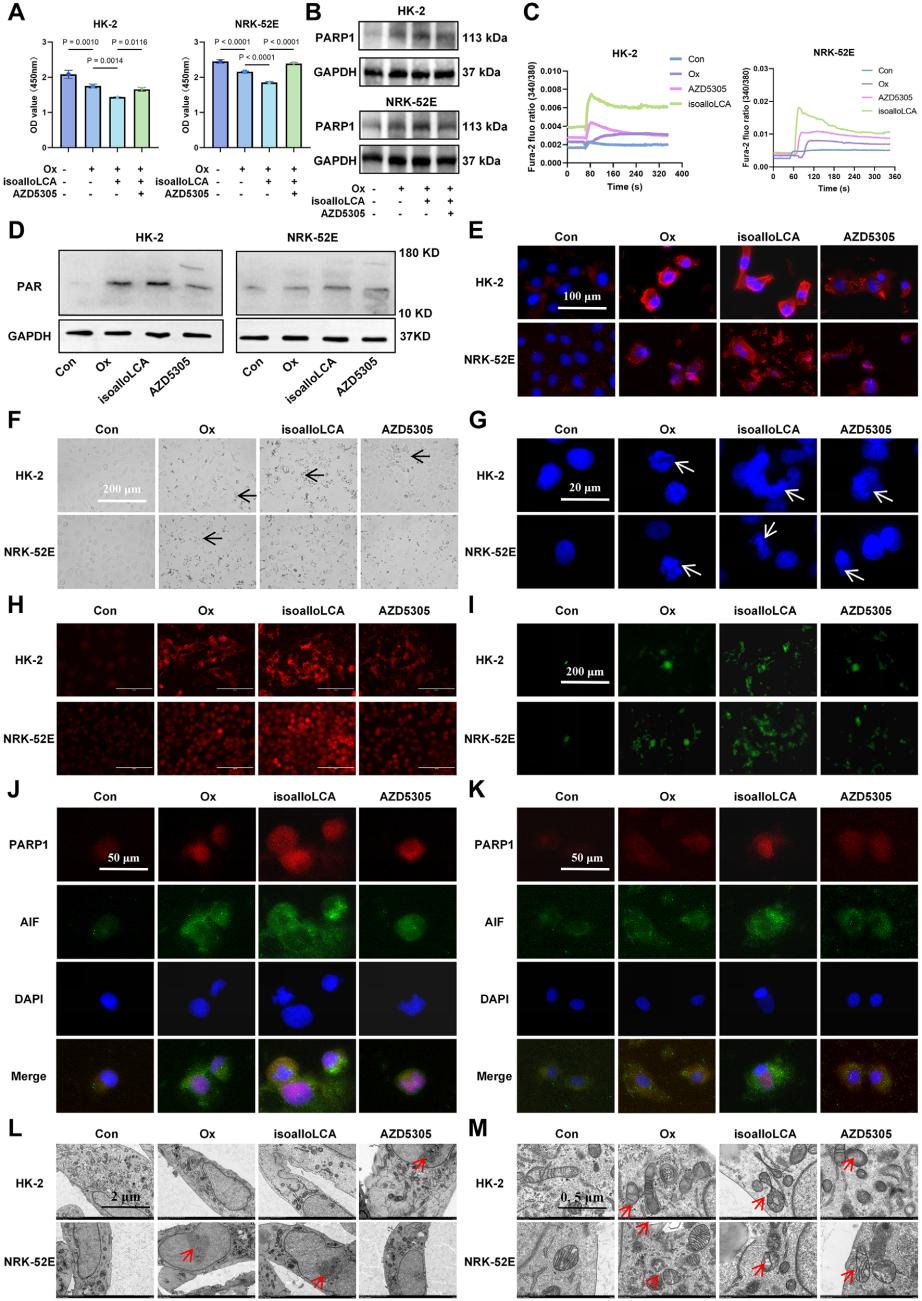
isoalloLCA induced PARP1‐mediated parthanatos in renal tubular epithelial cells. (A) The impact of isoalloLCA and PARP1 inhibitor on the viability of the oxalate‐induced cell models. (B) The effect of isoalloLCA and PARP1 inhibitor on PARP1 protein expression in the oxalate‐induced cell models. (C) The Ca^2+^ flux of different groups. (D‐E) Western blot (D) and immunofluorescence staining (E) of PAR in HK‐2 and NRK‐52E cells (scale bars, 100 µm). (F) Representative cell images under light microscope (arrows indicating oxalate crystals, scale bars, 200 µm). (G) DAPI staining of HK‐2 and NRK‐52E cells among different groups (arrows indicate chromatin condensation; scale bars, 20 µm). (H‐I) Representative DHE staining (H) and TUNEL staining (I) images under fluorescence microscope. (J‐K) Co‐localization of AIF (green) and PARP1 (red) in HK‐2 (J) and NRK‐52E (K) cells (scale bars, 50 µm). (L) Representative cell images in different groups under transmission electron microscope (arrows indicate chromatin condensation; scale bar, 2 µm; magnification, x 5000). (M) Representative cell images in different groups under transmission electron microscope (arrows indicate damaged mitochondria; scale bar, 0.5 µm; magnification, x 20000, higher magnification of Figure 10L ). (Con group: control; Ox group: oxalate; isoalloLCA group: oxalate+ isoalloLCA; AZD5305 group: oxalate+ isoalloLCA+AZD5305. Data are presented as mean ± SD, *n* = 3. *p*‐values were determined by one‐way ANOVA).

Light microscopy observed a significant increase in oxalate crystal deposition in the isoalloLCA group, but a decrease in the AZD5305 group (Figure 10F). DAPI staining revealed that isoalloLCA treatment induced marked chromatin condensation and nuclear fragmentation, characterized by bright, condensed nuclei and apoptotic bodies, which were reversed by AZD5305 co‐treatment (Figure 10G). DHE (Figure 10H) and TUNEL (Figure 10I) staining revealed that isoalloLCA treatment obviously enhanced ROS intensity and apoptosis cells, which was suppressed by the PARP1 inhibitor AZD5305. And the statistical analysis of DHE and TUNEL staining was shown in Figure S8D,E. Microscopy showed enhanced PARP1 expression and AIF nuclear translocation under oxalate and isoalloLCA, which was reversed by the PARP1 inhibitor AZD5305 in HK‐2 (Figure 10J) and in NRK‐52E cells (Figure 10K). Representative cell images (magnification, x 5000) in different groups under TEM were shown in Figure 10L. TEM of HK‐2 and NRK‐52E cells (magnification, x 20000) showed that isoalloLCA induced mitochondrial pathology, including swelling, cristae disruption, and increased membrane density, which were reversed by AZD5305 treatment, indicating PARP1‐mediated regulation of mitochondrial function (Figure 10M).

Upon isoalloLCA treatment, AIF and MIF protein levels were markedly increased in the nuclear fraction, whereas their levels were correspondingly decreased in the mitochondrial fraction, indicating their release from mitochondria and subsequent translocation into the nucleus of HK‐2 (Figure 11A) and NRK‐52E cells (Figure 11B). Consistently, immunofluorescence double staining revealed prominent colocalization of AIF and MIF within the nucleus of isoalloLCAtreated HK‐2 (Figure 11C) and NRK‐52E cells (Figure 11D). Cellular LDH, NAD^+^ and ATP levels were also measured. Following isoalloLCA exposure, a marked increase in LDH release was observed, indicating severe cell membrane damage, which was reversed by AZD5305 (Figure 11E). Following isoalloLCA exposure, a marked depletion of both ATP (Figure 11F) and NAD^+^ (Figure 11G) was observed, indicating severe energy crisis, which was reversed by AZD5305. Following oxalate and isoalloLCA treatment, a significant decrease was observed in Mito‐Tracker Red fluorescence intensity, indicating loss of mitochondrial membrane potential, along with increased Annexin V‐FITC positivity in HK‐2 (Figure S9A) and NRK‐52E cells (Figure S9B). These effects were reversed by cotreatment with the PARP1 inhibitor AZD5305. Furthermore, the activities of GSH‐Px, SOD, and MDA were evaluated. As shown in Figure S9C‐E, the levels of SOD and GSH‐Px were significantly elevated in the isoalloLCA group, but reduced in the AZD5305 group, whereas the MDA levels exhibited an opposite trend. These results reveal that isoalloLCA promoted CaOx stone formation via PARP1‐mediated parthanatos. The schematic diagram is proposed in Figure 11H.

**FIGURE 11:**
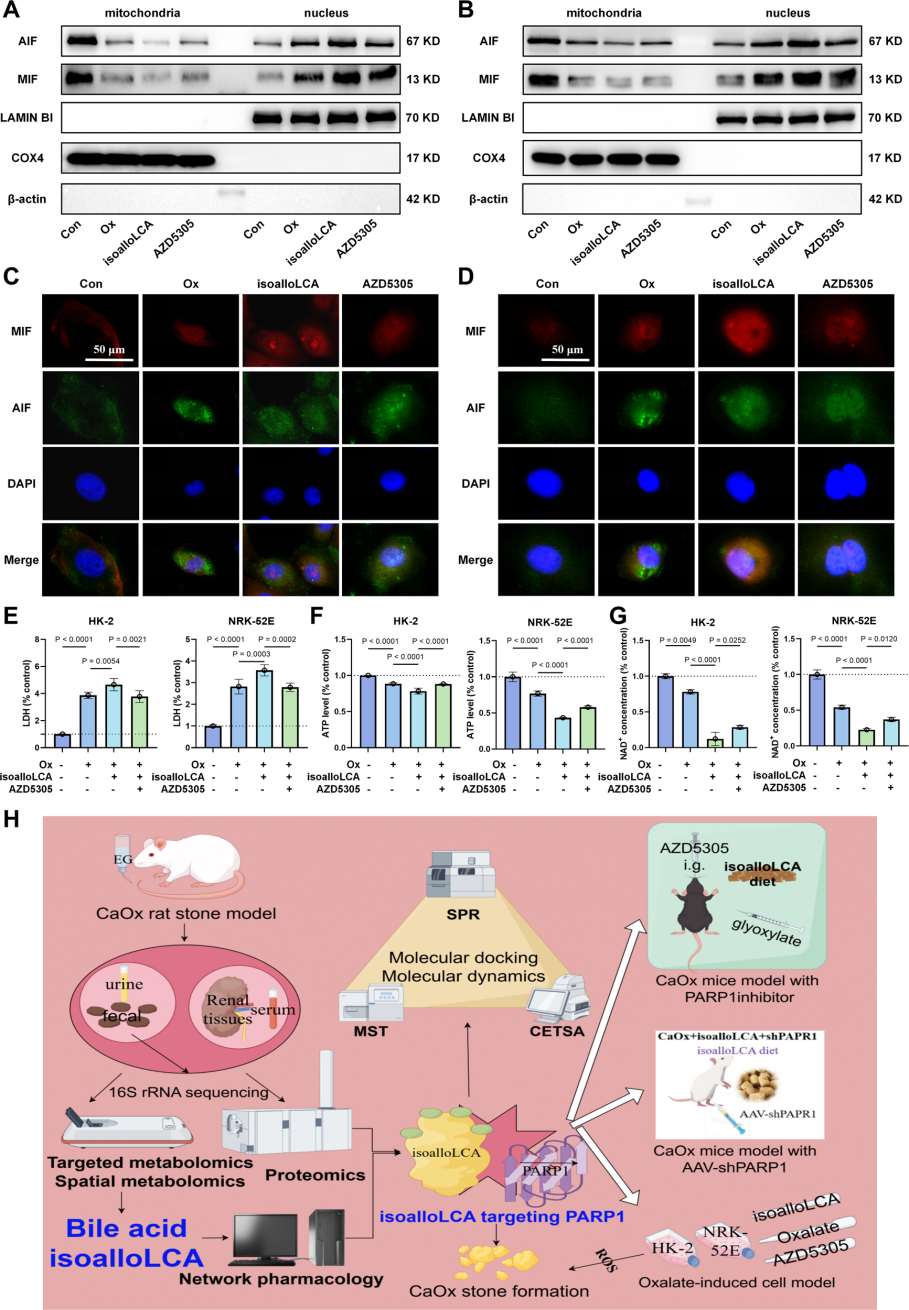
IsoalloLCA promotes CaOx stone formation via PARP1‐mediated parthanatos. (A‐B) Western blot analysis of AIF and MIF protein expression in mitochondrial and nuclear fractions of HK‐2 (A) and NRK‐52E cells (B). (C‐D) Co‐localization AIF (green) with MIF (red) in HK‐2 (C) and NRK‐52E (D) cells (scale bars, 50 µm). (E‐G) Cellular LDH (E), ATP (F), and NAD^+^ levels (G) in each group. (H) The mechanism diagram. (Con group: control; Ox group: oxalate; isoalloLCA group: oxalate+ isoalloLCA; AZD5305 group: oxalate+ isoalloLCA+AZD5305. Data are presented as mean ± SD, *n* = 3. *p*‐values were determined by one‐way ANOVA).

### 3.13 Exclusion of FXR Involvement in isoalloLCA‐mediated Effects in CaOx Nephrolithiasis

To assess the potential involvement of FXR in isoalloLCA‑mediated effects, we performed SPR analysis, which detected no direct binding between isoalloLCA and the FXR protein (Figure S10A). Functional validation was carried out in both HK‑2 and NRK‑52E cells. Treatment with isoalloLCA alone induced significant cell injury; however, co‑administration of the FXR antagonist UDCA (Figure S10B) or GS (Figure S10C), did not attenuate isoalloLCA‑induced cytotoxicity in HK‐2 and NRK‐52E cells. These findings collectively indicate that FXR does not mediate the pathogenic effects of isoalloLCA in CaOx nephrolithiasis.

## 4 Discussion

The pathogenesis of CaOx nephrolithiasis remains complex and is still an unresolved global health challenge [34]. With the advancement of metabolomics, an increasing number of metabolites have been identified as potential risk factors for CaOx kidney stone formation [11, 35]. However, the relationships between bile acids and CaOx stone development remain to be elucidated. In our study, we explored the metabolic mechanisms underlying CaOx stone formation, emphasizing the role of isoalloLCA in CaOx nephtolithiasis. Subsequent experiments confirmed the affinity between isoalloLCA and its target PARP1, and demonstrated that isoalloLCA promotes renal tubular epithelial cell injury and parthanatos via PARP1 in vivo and in vitro. Collectively, our findings suggest that isoalloLCA can promote CaOx nephrolithiasis formation targeting PARP1‐mediated parthanatos through the gut‐kidney axis.

In recent years, with the emergence of the gut‐kidney axis concept, increasing attention has been given to how disruptions in microbial composition and metabolites influence the development of kidney diseases [36, 37]. Additionally, studies have demonstrated a close association between specific gut microbial communities and kidney stones, primarily mediated through complex metabolic pathways like oxalate degradation [38, 39]. For example, *Oxalobacter formigenes* can interact with colonic epithelium to induce colonic oxalate secretion, thereby reducing urinary oxalate excretion and potentially decreasing the risk of stone formation [40, 41]. Our results suggest that in the CaOx group, the intestinal barrier was compromised, evidenced by epithelial damage, inflammation, and decreased expression of tight junction proteins. Near‐undetectable OCLN expression indicates severe tight junction disruption. Such disruption enhances paracellular oxalate permeability, as intestinal oxalate absorption is predominantly passive and paracellular [42]. Barrier dysfunction alone can induce hyperoxaluria, and synbiotic supplementation alleviates oxalate overload by restoring barrier integrity [43, 44]. This mechanism is clinically relevant in conditions with altered intestinal permeability, including inflammatory bowel disease [45] and intestinal bypass surgery [46].

16S rRNA sequencing further revealed gut microbiota differences in the CaOx group, characterized by reduced alpha diversity, and enrichment of *p_Proteobacteria* and *g_Shigella*. Notably, *p_Proteobacteria* and *g_Shigella* are known to be pathogenic gut bacteria that can damage intestinal epithelial cells and disrupt gut function [47]. Additionally, previous studies have indicated that gut microbiota can modulate bile acid synthesis through intestinal feedback mechanisms [48]. Bile acids are metabolized in the intestine by the gut microbiota into secondary bile acids, thus functioning as important mediators of metabolic homeostasis [49]. Several existing widely targeted metabolomics studies on CaOx kidney stones have identified differences in bile acid components within the CaOx group, but none of these studies have investigated the specific mechanisms involved [50, 51, 52].

Bile acids are not only essential for lipid digestion and absorption but also serve as ligands for multiple receptors and function as signaling molecules, playing vital roles in inflammation and immune responses [53]. The main receptors for bile acids involved include FXR, PXR, VDR, and the membrane‐bound G protein‐coupled receptor TGR5 [54, 55]. Through these receptors, bile acids can influence kidney health via complex signaling pathways [56, 57]. For example, activation of FXR with agonists such as obeticholic acid has been shown to suppress renal inflammation and oxidative stress in models of LPS‐induced acute kidney injury [58]. Additionally, TGR5 mediates mitochondrial biogenesis and helps prevent renal oxidative damage and lipid accumulation, thereby mitigating kidney disease associated with obesity and diabetes [59]. Our previous study has reported that bile acids may serve as biomarkers for CaOx rats, but the experimental validation remains to be conducted [18]. Similarly, recent studies have underscored the importance of bile acids as key mediators in CaOx stone pathogenesis via the gut–kidney axis. For instance, deoxycholic acid (DCA) has been implicated in promoting hyperoxaluria and renal tubular ferroptosis in mice on a high‐oxalate diet [60], while probiotic intervention with *L. plantarum* J‐15 has been shown to modulate secondary bile acid pathways and reduce stone formation, although the specific bile acids involved were not identified [50]. In this study, targeted metabolomics and spatial metabolomics analysis revealed distinct bile acid profiles and location between the control and CaOx groups. Moreover, consistent upregulation of isoalloLCA across rat stool, serum, kidneys, and urine underscored its role as a key mediator through the gut‐kidney axis.

However, research on isoalloLCA remains limited. Currently, the limited available evidence suggests that isoalloLCA, a derivative of lithocholic acid, enhances the differentiation of Treg cells, potentially through the stimulation of Foxp3 expression, a transcription factor essential for Treg cell development [61]. Additionally, studies have indicated that the nuclear hormone receptor NR4A1 is necessary for the influence of isoalloLCA on Treg cells [33]. In this study, we identify a previously unrecognized role of isoalloLCA in promoting CaOx stone formation, which may inform subsequent therapeutic strategies for CaOx stones. As a microbiota‐derived secondary bile acid, isoalloLCA is produced by *o_Bacteroidales*, including *f_Bacteroidaceae, f_Odoribacteraceae*, and *s_Parabacteroides merdae* [32, 49]. Its elevated levels in feces, plasma, urine, and kidneys of CaOx rats reflect systemic distribution through the gut–kidney axis: produced in the gut, absorbed into circulation, and excreted into urine for potential uptake by renal epithelial cells. Consistent, elevated isoalloLCA levels were detected in the CaOx group, along with increased abundance of *o_Bacteroidales* and its biosynthetic genes. Moreover, isoalloLCA positively correlated with *R. gnavus*, which can produce isobile acids via 3βHSDH [62], suggesting its potential role in isoalloLCA biosynthesis. Conversely, isoalloLCA negatively correlated with *F. prausnitzii*, and *F. prausnitzii* is linked to decreased secondary bile acids and BaiF/BaiH [60]. Together, these findings establish a robust correlative link between gut microbiota dysbiosis and elevated isoalloLCA. Furthermore, ABX‐mediated depletion of the gut microbiota markedly reduced isoalloLCA levels, whereas FMT from the Gly mice donors restored the elevation of isoalloLCA and recapitulated the stone phenotype, as confirmed by 16S rRNA sequencing and targeted metabolomic analyses. These results collectively demonstrate a causal link between gut microbiota dysbiosis and altered isoalloLCA metabolism, establishing that the gut microbiome drives the production of isoalloLCA and subsequent CaOx stone formation.

By combining proteomics with network pharmacology, we identified PARP1 as the key target of isoalloLCA in CaOx stone formation. The gene PARP1 encodes a chromatin‐associated enzyme, poly(ADP‐ribose) polymerase, functioning in DNA repair, stabilization of DNA replication forks, and modulation of chromatin structure [63]. It has also been demonstrated that the activity of PARP enzymes is closely associated with bile acid and lipid metabolism [64, 65]. A study has shown that PARP1 is overexpressed in response to acidic bile reflux, and PARP1 serves as an important early responder to DNA damage [65]. Moreover, we confirmed that isoalloLCA directly binds PARP1 (Kd = 4.31–8.0 µm). Furthermore, isoalloLCA appears to promote cell damage through PARP1‐mediated oxidative stress (increased SOD and GSH‐Px activity and decreased MDA), yet the specific mechanisms that warrant further study.

Given that AAV‐shPARP1 and the PARP1 inhibitor (AZD5305) blocked isoalloLCA‐mediated ROS elevation, mitochondrial damage, and crystal deposition in the CaOx rats and mice, we hypothesize that this process may be related to PARP1‐dependent cell death, known as parthanatos. The mechanisms of parthanatos primarily include PARP1 hyper‐activation, DNA damage, accumulation of poly (ADP‐ribose), depletion of NAD and ATP, and translocation of AIF and MIF to the nucleus [29, 30, 66]. Furthermore, the in vitro assays revealed that excessive PARP1 activation, and nuclear translocation of AIF occurred in the oxalate and the isoalloLCA groups. So far, parthanatos has not been reported in CaOx nephrolithiasis; however, parthanatos has been implicated in pathological cell injury associated with renal diseases, and ischemia‐reperfusion injury [67]. In a mouse model of renal ischemia‐reperfusion injury, PARP1 inhibition reduced necrosis and enhanced tissue recovery [68]. Parthanatos is strictly dependent on PARP1 hyperactivation, distinguishing it from other cell death pathways where PARP1 is only indirectly involved [69]. We prioritized parthanatos because our proteomic data revealed PARP1‐centered responses, and follow‐up experiments confirmed its hallmark features—PAR accumulation, AIF and MIF nuclear translocation—providing a mechanistic link between isoalloLCA‐induced injury and this specific cell death pathway. PARP1 and parthanatos‐induced tubular cell death might generate cellular debris that serves as a nidus for crystal nucleation and aggregation [70]. Concurrently, damage‐associated molecular patterns (HMGB1 and ATP) released from dying cells amplify local inflammation and create a procalcific microenvironment, driving a selfperpetuating cycle of injury and crystal formation [71]. Therefore, targeting PARP1 and parthanatos may represent a promising strategy for preventing or alleviating kidney stone formation, although further validation is required. AZD5305 is a next‐generation PARP1‐selective inhibitor designed to overcome the hematological toxicity of first‐generation PARP1/2 inhibitors by sparing PARP2 [72]. These findings support its favorable safety profile and therapeutic potential for kidney stone disease.

Our study integrates multi‐omics (microbiome, metabolomics, proteomics, network pharmacology) and biophysical assays (SPR, MST, CETSA, molecular docking, MD simulation) to elucidate the isoalloLCA‐PARP1 axis in CaOx nephrolithiasis via the gut‐kidney axis across the CaOx rat, mouse, and cell models. Moreover, our investigations demonstrated that isoalloLCA promotes CaOx kidney stone formation via PARP1‐mediated parthanatos, providing the first evidence implicating parthanatos in CaOx nephrolithiasis and identifying isoalloLCA–PARP1 as a pathogenic, actionable target. However, there are limitations to this study: 1. Our experiments were conducted mainly in animal and cell models, and the small clinical sample size limits the generalizability of our findings. 2. We used only AAV and pharmacological inhibitors of PARP1 in this study, but gene knockout of PARP1 in animals or cells was not employed. 3. The mechanistic depth is limited, and the downstream signaling pathways require further investigation to elucidate the complete molecular mechanisms involved. 4. The definitive source attribution of isoalloLCA will require antibiotic/germ‐free validation, fecal microbiota transfer, and in vitro substrate‐conversion assays coupled with mass spectrometry. 5. PARP1 mainly exists in nucleus, but the direct visualization of isoalloLCA‐PARP1 nuclear co‐localization is currently lacking in this study. Fluorescent isoalloLCA probes in future studies will further confirm its nuclear co‑localization.

## 5 Conclusion

This multi‐omics study reveals that isoalloLCA promotes CaOx kidney stone formation by binding to and activating PARP1, thereby inducing parthanatos in renal tubular epithelial cells. These findings not only establish isoalloLCA–PARP1 axis as a key pathogenic mechanism in CaOx stone formation, but also provide a mechanistic basis for targeting parthanatos as a therapeutic strategy in CaOx nephrolithiasis.

## Author Contributions

ZZJ designed the study, performed the experiments and drafted the manuscript. YRX, ZX, and SF performed the experiments and conducted the analyses. LZY and GP collected the data. WLJ, WZ, WDW, and FNH designed and supervised the study and revised the manuscript.

## Funding

This research was funded by the National Natural Science Foundation of China (No. 82500928 and No. 82671010).

## Ethics Approval and Patient Consent Statement

Ethical approval of this study was granted by the Huashan Institutional Review Board of Fudan University (HIRB, 2025‐1300). Participants gave informed consent to participate in the study before taking part. The Fudan Laboratory Animal Ethics Board approved this animal study (2025‐HSYY‐557). Written informed consent was provided from all the patients.

## Conflicts of Interest

The authors declare no conflicts of interests.

## Supporting information




**Supporting File 1**: advs77289‐sup‐0001‐SuppMat.docx.

**Supporting File 2**: advs77289‐sup‐0002‐SuppMat.xlsx.

## Data Availability

The data that support the findings of this study are available from the corresponding author upon reasonable request.

## References

[1] M. Bargagli , M. Scoglio , S. A. Howles , and D. G. Fuster , “Kidney Stone Disease: Risk Factors, Pathophysiology and Management,” Nature Reviews Nephrology 21 (2025): 794–808. 10.1038/s41581-025-00990-x 40790363

[2] C. Thongprayoon , A. E. Krambeck , and A. D. Rule , “Determining the True Burden of Kidney Stone Disease,” Nature Reviews Nephrology 16, no. 12 (2020): 736–746. 10.1038/s41581-020-0320-7 32753740

[3] P. Peerapen and V. Thongboonkerd , “Kidney Stone Prevention,” Advances in Nutrition 14, no. 3 (2023): 555–569. 10.1016/j.advnut.2023.03.002 PMC10201681 36906146

[4] S. A. Howles and R. V. Thakker , “Genetics of Kidney Stone Disease,” Nature Reviews Urology 17, no. 7 (2020): 407–421. 10.1038/s41585-020-0332-x 32533118

[5] S. R. Khan , B. K. Canales , and P. R. Dominguez‐Gutierrez , “Randall's Plaque and Calcium Oxalate Stone Formation: Role for Immunity and Inflammation,” Nature Reviews Nephrology 17, no. 6 (2021): 417–433. 10.1038/s41581-020-00392-1 33514941

[6] X. Duan , Z. Kong , X. Mai , et al., “Autophagy Inhibition Attenuates Hyperoxaluria‐induced Renal Tubular Oxidative Injury and Calcium Oxalate Crystal Depositions in the Rat Kidney,” Redox Biology 16 (2018): 414–425. 10.1016/j.redox.2018.03.019 PMC5953241 29653411

[7] S. A. Doumas , C. Tsironis , A. A. Bolaji , P. Garantziotis , and E. Frangou , “Glomerulonephritis and Inflammatory Bowel Disease: a Tale of Gut‐kidney Axis Dysfunction,” Autoimmunity Reviews 22, no. 6 (2023): 103327. 10.1016/j.autrev.2023.103327 36990134

[8] A. Ticinesi , C. Milani , A. Guerra , et al., “Understanding the Gut–kidney Axis in Nephrolithiasis: an Analysis of the Gut Microbiota Composition and Functionality of Stone Formers,” Gut 67, no. 12 (2018): 2097–2106. 10.1136/gutjnl-2017-315734 29705728

[9] S. Robijn , B. Hoppe , B. A. Vervaet , P. C. D'Haese , and A. Verhulst , “Hyperoxaluria: a Gut–kidney Axis?,” Kidney International 80, no. 11 (2011): 1146–1158. 10.1038/ki.2011.287 21866092

[10] Y. Liu , X. Jin , Y. Ma , et al., “Short‐Chain Fatty Acids Reduced Renal Calcium Oxalate Stones by Regulating the Expression of Intestinal Oxalate Transporter SLC26A6,” mSystems 6, no. 6 (2021): 0104521. 10.1128/mSystems.01045-21 PMC8594443 34783577

[11] X. Z. Zhang , X. X. Lei , Y. L. Jiang , et al., “Application of Metabolomics in Urolithiasis: the Discovery and Usage of succinate,” Signal Transduction and Targeted Therapy 8, no. 1 (2023): 41. 10.1038/s41392-023-01311-z PMC9867757 36681678

[12] P. M. Ferraro , Y. Li , R. Balasubramanian , G. C. Curhan , and E. N. Taylor , “The Plasma Metabolome and Risk of Incident Kidney Stones,” Journal of the American Society of Nephrology 35, no. 10 (2024): 1412–1421. 10.1681/ASN.0000000000000421 PMC11452138 38865256

[13] Q. Nie , X. Luo , K. Wang , et al., “Gut Symbionts Alleviate MASH through a Secondary Bile Acid Biosynthetic Pathway,” Cell 187, no. 11 (2024): 2717–2734.e33. 10.1016/j.cell.2024.03.034 38653239

[14] S. Lin , S. Wang , P. Wang , et al., “Bile Acids and Their Receptors in Regulation of Gut Health and Diseases,” Progress in Lipid Research 89 (2023): 101210. 10.1016/j.plipres.2022.101210 36577494

[15] Y. Zhuang , M. Ortega‐Ribera , P. Thevkar Nagesh , et al., “Bile Acid–induced IRF3 Phosphorylation Mediates Cell Death, Inflammatory Responses, and Fibrosis in Cholestasis‐induced Liver and Kidney Injury via Regulation of ZBP1,” Hepatology 79, no. 4 (2024): 752–767. 10.1097/HEP.0000000000000611 PMC10948324 37725754

[16] B. Guan , J. Tong , H. Hao , et al., “Bile Acid Coordinates Microbiota Homeostasis and Systemic Immunometabolism in Cardiometabolic Diseases,” Acta Pharmaceutica Sinica B 12, no. 5 (2022): 2129–2149. 10.1016/j.apsb.2021.12.011 PMC9136572 35646540

[17] J. W. Dobbins and H. J. Binder , “Effect of Bile Salts and Fatty Acids on the Colonic Absorption of Oxalate,” Gastroenterology 70, no. 6 (1976): 1096–1100. 1269869

[18] Z. Zhou , D. Feng , D. Shi , P. Gao , L. Wang , and Z. Wu , “Untargeted and Targeted Metabolomics Reveal Bile Acid Profile Changes in Rats with Ethylene Glycol‐induced Calcium Oxalate Nephrolithiasis,” Chemico‐Biological Interactions 381 (2023): 110570. 10.1016/j.cbi.2023.110570 37244400

[19] K. Kim , T. Thome , C. Pass , et al., “Multiomic Analysis of Calf Muscle in Peripheral Artery Disease and Chronic Kidney Disease,” Circulation Research 136, no. 7 (2025): 688–703. 10.1161/CIRCRESAHA.124.325642 PMC11949227 39963788

[20] V. H. Canela , W. S. Bowen , R. M. Ferreira , et al., “A Spatially Anchored Transcriptomic Atlas of the human Kidney Papilla Identifies Significant Immune Injury in Patients with Stone Disease,” Nature Communications 14, no. 1 (2023): 4140. 10.1038/s41467-023-38975-8 PMC10356953 37468493

[21] C. Shi , D. Zhao , J. Butler , et al., “Multi‐omics Analysis in Primary T Cells Elucidates Mechanisms behind Disease‐associated Genetic Loci,” Genome Biology 26, no. 1 (2025): 26. 10.1186/s13059-025-03492-y PMC11808986 39930543

[22] R. F. Dubin and E. P. Rhee , “Proteomics and Metabolomics in Kidney Disease, Including Insights into Etiology, Treatment, and Prevention,” Clinical Journal of the American Society of Nephrology 15, no. 3 (2020): 404–411. 10.2215/CJN.07420619 PMC7057308 31636087

[23] C. Nogales , Z. M. Mamdouh , M. List , C. Kiel , A. I. Casas , and H. Schmidt , “Network Pharmacology: Curing Causal Mechanisms Instead of Treating Symptoms,” Trends in Pharmacological Sciences 43, no. 2 (2022): 136–150. 10.1016/j.tips.2021.11.004 34895945

[24] S. Yoodee , P. Peerapen , P. Rattananinsruang , S. Detsangiamsak , S. Sukphan , and V. Thongboonkerd , “Large‐scale Identification of Calcium Oxalate Stone Inhibitory Proteins in Normal human Urine,” International Journal of Biological Macromolecules 275, no. Pt 2 (2024): 133646. 10.1016/j.ijbiomac.2024.133646 38969041

[25] H. Li , F. Xu , C. Liu , et al., “Inhibitory Effects and Surface Plasmon Resonance‐Based Binding Affinities of Dietary Hydrolyzable Tannins and Their Gut Microbial Metabolites on SARS‐CoV‐2 Main Protease,” Journal of Agricultural and Food Chemistry 69, no. 41 (2021): 12197–12208. 10.1021/acs.jafc.1c03521 PMC8491554 34586788

[26] G. Illuzzi , A. D. Staniszewska , S. J. Gill , et al., “Preclinical Characterization of AZD5305, a Next‐Generation, Highly Selective PARP1 Inhibitor and Trapper,” Clinical Cancer Research 28, no. 21 (2022): 4724–4736. 10.1158/1078-0432.CCR-22-0301 PMC9623235 35929986

[27] A. Herencia‐Ropero , A. Llop‐Guevara , A. D. Staniszewska , et al., “The PARP1 Selective Inhibitor saruparib (AZD5305) Elicits Potent and Durable Antitumor Activity in Patient‐derived BRCA1/2‐associated Cancer Models,” Genome Medicine 16, no. 1 (2024): 107. 10.1186/s13073-024-01370-z PMC11348616 39187844

[28] Z. Zhou , X. Zhou , Y. Zhang , Y. Yang , L. Wang , and Z. Wu , “Butyric Acid Inhibits Oxidative Stress and Inflammation Injury in Calcium Oxalate Nephrolithiasis by Targeting CYP2C9,” Food and Chemical Toxicology 178 (2023): 113925. 10.1016/j.fct.2023.113925 37414240

[29] L. Zhu , Z. Xie , G. Yang , G. Zhou , L. Li , and S. Zhang , “Stanniocalcin‐1 Promotes PARP1‐Dependent Cell Death via JNK Activation in Colitis,” Advanced Science 11, no. 5 (2024): 2304123. 10.1002/advs.202304123 PMC10837357 38088577

[30] J. Zhang , X. Hu , Y. Geng , et al., “Exploring the Role of Parthanatos in CNS Injury: Molecular Insights and Therapeutic Approaches,” Journal of Advanced Research 70 (2025): 271–286. 10.1016/j.jare.2024.04.031 PMC11976428 38704090

[31] W. Huang , L. Zhou , H. Guo , Y. Xu , and Y. Xu , “The Role of Short‐chain Fatty Acids in Kidney Injury Induced by Gut‐derived Inflammatory Response,” Metabolism 68 (2017): 20–30. 10.1016/j.metabol.2016.11.006 28183450

[32] Y. Sato , K. Atarashi , D. R. Plichta , et al., “Novel Bile Acid Biosynthetic Pathways Are Enriched in the Microbiome of Centenarians,” Nature 599, no. 7885 (2021): 458–464. 10.1038/s41586-021-03832-5 34325466

[33] W. Li , S. Hang , Y. Fang , et al., “A Bacterial Bile Acid Metabolite Modulates Treg Activity through the Nuclear Hormone Receptor NR4A1,” Cell Host & Microbe 29, no. 9 (2021): 1366–1377.e9. 10.1016/j.chom.2021.07.013 PMC9064000 34416161

[34] S. Shastri , J. Patel , K. K. Sambandam , and E. D. Lederer , “Kidney Stone Pathophysiology, Evaluation and Management: Core Curriculum 2023,” American Journal of Kidney Diseases 82, no. 5 (2023): 617–634. 10.1053/j.ajkd.2023.03.017 PMC11370273 37565942

[35] N. S. Kowalczyk , M. L. Prochaska , and E. M. Worcester , “Metabolomic Profiles and Pathogenesis of Nephrolithiasis,” Current Opinion in Nephrology & Hypertension 32, no. 5 (2023): 490–495. 10.1097/MNH.0000000000000903 PMC10403267 37530089

[36] Q. Fu , Y. Yang , Q. Tian , et al., “Exploring the Mechanism of Paotianxiong Polysaccharide in the Treatment of Chronic Kidney Disease Combining Metabolomics and Microbiomics Technologies,” International Journal of Biological Macromolecules 289 (2025): 138629. 10.1016/j.ijbiomac.2024.138629 39667450

[37] Y. Zhang , J. Men , K. Yin , et al., “Activation of Gut Metabolite ACSL4/LPCAT3 by Microplastics in Drinking Water Mediates Ferroptosis via Gut–kidney Axis,” Communications Biology 8, no. 1 (2025): 211. 10.1038/s42003-025-07641-8 PMC11811008 39930042

[38] B. Hussain , C. C. Wu , H. C. Tsai , et al., “Species‐level Characterization of Gut Microbiota and Their Metabolic Role in Kidney Stone Formation Using Full‐length 16S rRNA Sequencing,” Urolithiasis 52, no. 1 (2024): 115. 10.1007/s00240-024-01610-2 39126448

[39] Y. Liu , X. Jin , H. G. Hong , et al., “The Relationship between Gut Microbiota and Short Chain Fatty Acids in the Renal Calcium Oxalate Stones Disease,” The FASEB Journal 34, no. 8 (2020): 11200–11214. 10.1096/fj.202000786R 32645241

[40] D. Arvans , Y. C. Jung , D. Antonopoulos , et al., “Oxalobacter Formigenes–Derived Bioactive Factors Stimulate Oxalate Transport by Intestinal Epithelial Cells,” Journal of the American Society of Nephrology 28, no. 3 (2017): 876–887. 10.1681/ASN.2016020132 PMC5328155 27738124

[41] M. Liu , H. Koh , Z. D. Kurtz , et al., “Oxalobacter Formigenes‐associated Host Features and Microbial Community Structures Examined Using the American Gut Project,” Microbiome 5, no. 1 (2017): 108. 10.1186/s40168-017-0316-0 PMC5571629 28841836

[42] J. M. Whittamore and M. Hatch , “Oxalate Flux across the Intestine: Contributions from Membrane Transporters,” Comprehensive Physiology 12, no. 1 (2021): 2835–2875. 10.1002/cphy.c210013 34964122

[43] H. A. Hassan , M. E. Bashir , A. Alshaikh , D. Y. Jung , J. R. Turner , and J. R. Asplin , Journal of the American Society of Nephrology 32, no. 10S (2021): 203.

[44] X. M. Li , Y. Amier , W. L. Wan , et al., “A Novel Synbiotic Formulation Prevents Calcium Oxalate Stones by Restoring Gut Microbiota Homeostasis,” BMC Microbiology 25, no. 1 (2025): 673. 10.1186/s12866-025-04408-3 PMC12539186 41120928

[45] J. W. Dobbins , “Nephrolithiasis and Intestinal Disease,” Journal of Clinical Gastroenterology 7, no. 1 (1985): 21–24. 10.1097/00004836-198502000-00002 3980960

[46] J. Q. Stauffer , “Hyperoxaluria and Calcium Oxalate Nephrolithiasis after Jejunoileal Bypass,” The American Journal of Clinical Nutrition 30, no. 1 (1977): 64–71. 10.1093/ajcn/30.1.64 831440

[47] A. Grassart , V. Malarde , S. Gobaa , et al., “Bioengineered Human Organ‐on‐Chip Reveals Intestinal Microenvironment and Mechanical Forces Impacting Shigella Infection,” Cell Host & Microbe 26, no. 4 (2019): 565. 10.1016/j.chom.2019.09.007 31600505

[48] L. Zhao , W. Yang , Y. Chen , et al., “A Clostridia‐rich Microbiota Enhances Bile Acid Excretion in Diarrhea‐predominant Irritable Bowel Syndrome,” Journal of Clinical Investigation 130, no. 1 (2020): 438–450. 10.1172/JCI130976 PMC6934182 31815740

[49] D. Paik , L. Yao , Y. Zhang , et al., “Human Gut Bacteria Produce ΤΗ17‐modulating Bile Acid Metabolites,” Nature 603, no. 7903 (2022): 907–912. 10.1038/s41586-022-04480-z PMC9132548 35296854

[50] L. Tian , Y. Liu , X. Xu , et al., “Lactiplantibacillus Plantarum J‐15 Reduced Calcium Oxalate Kidney Stones by Regulating Intestinal Microbiota, Metabolism, and Inflammation in Rats,” The FASEB Journal 36, no. 6 (2022): 22340. 10.1096/fj.202101972RR 35524736

[51] H. Xie , R. Wang , L. Xie , X. Wang , and C. Liu , “Study on the Pathogenesis and Prevention Strategies of Kidney Stones Based on GC–MS Combined with Metabolic Pathway Analysis,” Rapid Communications in Mass Spectrometry 36, no. 22 (2022): 9387. 10.1002/rcm.9387 36039746

[52] S. Gao , R. Yang , Z. Peng , et al., “Metabolomics Analysis for Hydroxy‐L‐proline‐induced Calcium Oxalate Nephrolithiasis in Rats Based on Ultra‐high Performance Liquid Chromatography Quadrupole Time‐of‐flight Mass Spectrometry,” Scientific Reports 6, no. 1 (2016): 30142. 10.1038/srep30142 PMC4957101 27443631

[53] C. D. Fuchs , B. Simbrunner , M. Baumgartner , C. Campbell , T. Reiberger , and M. Trauner , “Bile Acid Metabolism and Signalling in Liver Disease,” Journal of Hepatology 82, no. 1 (2025): 134–153. 10.1016/j.jhep.2024.09.032 39349254

[54] A. Perino , H. Demagny , L. Velazquez‐Villegas , and K. Schoonjans , “Molecular Physiology of Bile Acid Signaling in Health, Disease, and Aging,” Physiological Reviews 101, no. 2 (2021): 683–731. 10.1152/physrev.00049.2019 32790577

[55] M. M. Thibaut and L. B. Bindels , “Crosstalk between Bile Acid‐activated Receptors and Microbiome in Entero‐hepatic Inflammation,” Trends in Molecular Medicine 28, no. 3 (2022): 223–236. 10.1016/j.molmed.2021.12.006 35074252

[56] P. Malko and L. H. Jiang , “TRPM2 channel‐mediated Cell Death: an Important Mechanism Linking Oxidative Stress‐inducing Pathological Factors to Associated Pathological Conditions,” Redox Biology 37 (2020): 101755. 10.1016/j.redox.2020.101755 PMC7600390 33130440

[57] S. Yu , X. Gu , Q. Zheng , et al., “Tauroursodeoxycholic Acid Ameliorates Renal Injury Induced by COL4A3 Mutation,” Kidney International 106, no. 3 (2024): 433–449. 10.1016/j.kint.2024.04.015 PMC11343663 38782199

[58] J. B. Zhu , S. Xu , J. Li , et al., “Farnesoid X Receptor Agonist Obeticholic Acid Inhibits Renal Inflammation and Oxidative Stress during Lipopolysaccharide‐induced Acute Kidney Injury,” European Journal of Pharmacology 838 (2018): 60–68. 10.1016/j.ejphar.2018.09.009 30196109

[59] X. X. Wang , M. H. Edelstein , U. Gafter , et al., “G Protein‐Coupled Bile Acid Receptor TGR5 Activation Inhibits Kidney Disease in Obesity and Diabetes,” Journal of the American Society of Nephrology 27, no. 5 (2016): 1362–1378. 10.1681/ASN.2014121271 PMC4849814 26424786

[60] L. Liu , Y. Ma , Z. Jian , et al., “Gut Microbiota‐bile Acid Crosstalk Contributes to Calcium Oxalate Nephropathy through Hsp90α‐mediated Ferroptosis,” Cell Reports 44, no. 7 (2025): 115936. 10.1016/j.celrep.2025.115936 40591459

[61] S. Hang , D. Paik , L. Yao , et al., “Bile Acid Metabolites Control TH17 and Treg Cell Differentiation,” Nature 576, no. 7785 (2019): 143–148. 10.1038/s41586-019-1785-z PMC6949019 31776512

[62] A. S. Devlin and M. A. Fischbach , “A Biosynthetic Pathway for a Prominent Class of Microbiota‐derived Bile Acids,” Nature Chemical Biology 11, no. 9 (2015): 685–690. 10.1038/nchembio.1864 PMC4543561 26192599

[63] A. R. Chaudhuri and A. Nussenzweig , “The Multifaceted Roles of PARP1 in DNA Repair and Chromatin Remodelling,” Nature Reviews Molecular Cell Biology 18, no. 10 (2017): 610–621. 10.1038/nrm.2017.53 PMC6591728 28676700

[64] M. Szanto , R. Gupte , W. L. Kraus , P. Pacher , and P. Bai , “PARPs in Lipid Metabolism and Related Diseases,” Progress in Lipid Research 84 (2021): 101117. 10.1016/j.plipres.2021.101117 34450194

[65] P. G. Doukas , D. P. Vageli , and B. L. Judson , “The Role of PARP ‐1 and NF‐κB in Bile‐Induced DNA Damage and Oncogenic Profile in Hypopharyngeal Cells,” The Laryngoscope 133, no. 5 (2023): 1146–1155. 10.1002/lary.30284 35791892

[66] Y. Wang , R. An , G. K. Umanah , et al., “A Nuclease That Mediates Cell Death Induced by DNA Damage and Poly(ADP‐ribose) Polymerase‐1,” Science 354, no. 6308 (2016): 6308. 10.1126/science.aad6872 PMC5134926 27846469

[67] H. Zhao , J. Ning , A. Lemaire , et al., “Necroptosis and Parthanatos Are Involved in Remote Lung Injury after Receiving Ischemic Renal Allografts in Rats,” Kidney International 87, no. 4 (2015): 738–748. 10.1038/ki.2014.388 25517913

[68] J. Nehme , L. Mesilmany , M. Varela‐Eirin , et al., “Converting Cell Death into Senescence by PARP1 Inhibition Improves Recovery from Acute Oxidative Injury,” Nature Aging 4, no. 6 (2024): 771–782. 10.1038/s43587-024-00627-x 38724734

[69] R. D. Moura , P. D. Mattos , P. F. Valente , and N. C. Hoch , “Molecular Mechanisms of Cell Death by Parthanatos: More Questions than Answers,” Genetics and Molecular Biology 47, no. 1 (2024): 20230357. 10.1590/1678-4685-GMB-2023-0357 PMC11445734 39356140

[70] S. R. Mulay , J. Desai , S. V. Kumar , et al., “Cytotoxicity of Crystals Involves RIPK3‐MLKL‐mediated Necroptosis,” Nature Communications 7, no. 1 (2016): 10274. 10.1038/ncomms10274 PMC4738349 26817517

[71] S. R. Mulay and H. J. Anders , “Crystal Nephropathies: Mechanisms of Crystal‐induced Kidney Injury,” Nature Reviews Nephrology 13, no. 4 (2017): 226–240. 10.1038/nrneph.2017.10 28218266

[72] Y. Luo , M. Nie , Y. Chen , et al., “Clinical Application of PARP1 Inhibitors and Challenges in Cancer Therapy,” Current Cancer Drug Targets 26, no. 1 (2026): 1–35. 10.2174/0115680096327635241021115944 39779573

