# Supplementary material for: Gut Microbiota‐Isoallolithocholic Acid Crosstalk Promotes Calcium Oxalate Kidney Stone Formation via PARP1‐Mediated Parthanatos
Source: Adv Sci (Weinh). 2026 Aug 28;13(63):e77289. doi: 10.1002/advs.77289 (PMC13525351; doi:10.1002/advs.77289)
Supplement: Supplementary file 1 [file ADVS-13-e77289-s001.docx]

**Supplementary Figures**

**Figure S1. The bile acid metabolomics and 16S rRNA sequencing analysis of rats from different groups.** (A) The PCA plots of bile acid metabolomics in rat stool, serum, kidneys, and urine samples. (B) Species annotation at phylum and class levels. (C) Alpha diversity of the gut microbiota. (D) Spearman correlation analysis of *o_Bacteroidales* with isoalloLCA in kidney and urine.

**Figure S2. Proteomics analysis of rat serum and kidneys.** (A-B) The heatmap (A) and KEGG (B) enrichment analysis of differentially expressed proteins (DEPs) in serum between Con and CaOx groups. (C) GSEA analysis of DEPs in serum. (D-E) The heatmap (D) and PCA plot (E) of DEPs in kidneys between Con and CaOx groups. (F) GSEA analysis of DEPs in kidneys.

**Figure S3. isoalloLCA binds potently to PARP1.** (A) Microscale thermophoresis (MST) analysis of isoalloLCA binding to PARP1. (B-C) The radius of gyration graph (B) and surface analysis (C) of isoalloLCA with PARP1.

**Figure S4. Immunofluorescence of PARP1 in the intestinal epithelium of rats.**

**Figure S5. isoalloLCA promotes stone formation via PARP1 in CaOx mice.** (A-B) PAPR1 inhibitor blocked the isoalloLCA-mediated aggravation of weight loss (A) and serum inflammatory factors (B) in the CaOx mice. (C) The full-field Von Kossa staining image of each group of mice.

**Figure S6. PARP1 inhibition attenuates crystal deposition and renal injury in glyoxylic acid-induced CaOx mice.** (A) Schematic diagram of the experimental design (Con: saline i.p. + saline gavage, AZD5305 alone: saline i.p. + AZD5305 gavage, GLy alone: glyoxylic acid i.p. + saline gavage, and Gly + AZD5305: glyoxylic acid i.p. + AZD5305 gavage). (B) Body weight changes during the treatment period. (C) Representative H&E and Von Kossa (VK) staining of kidney sections (arrows indicate crystal deposition and tubular injury). (D) Quantitative analysis of the number of H&E and VK staining. (E) Renal function assessment in each group. (F) Serum inflammatory factors in each group using ELISA. (Data are presented as mean ± SD, n=5)

**Figure S7. PARP1-induced Parthanatos promoted cell injury in renal tubular epithelial cells.** (A) The PARP1 expression protein after transfected with PARP1 overexpression lentivirus or negative control (NC). (B) The impact of PARP1 on the viability of HK-2 and NRK-52E cells. (C-D) Microscopy showing AIF (green) co-localization with PARP1 (red) in HK-2 (C) and NRK-52E (D) cells. Scale bars, 50 μm. (E) PARP1 promote oxalate-induced cell injury in renal tubular epithelial cells.

**Figure S8. IsoalloLCA promotes cell injury via PARP1.** (A) The effect of different concentrations of isoalloLCA on the viability of HK-2 and NRK-52E cells. (B-C) Immunofluorescence analysis of PAR and γ-H2AX in CaOx mice (B) and rats (C). (D-E) Statistical analysis of DHE (D) and TUNEL staining (E) in HK-2 and NRK-52E cells.

**Figure S9. IsoalloLCA promotes mitochondrial dysfunction in HK-2 and NRK-52E cells.** (A-B) Mito-Tracker Red CMXRos and Annexin V-FITC double staining in HK-2 (A) and NRK-52E cells (B). (C-E) The activities of SOD (C), MDA (D), and GSH-Px (E) in different groups.

**Figure S10. Exclusion of FXR involvement in isoalloLCA‑mediated effects in CaOx nephrolithiasis.** (A) Surface plasmon resonance (SPR) analysis between isoalloLCA and the FXR protein. (B-C) Co‑administration of the FXR antagonist UDCA (B) or GS (C) did not attenuate isoalloLCA‑induced cytotoxicity (GS: Guggulsterone, UDCA: Ursodeoxycholic acid).
